# Enhanced Compound Analysis Using Reactive Paper Spray Mass Spectrometry: Leveraging Schiff Base Reaction for Amino Acid Detection

**DOI:** 10.1021/acs.analchem.4c00215

**Published:** 2024-03-20

**Authors:** Marcos Bouza, Daniel Foest, Sebastian Brandt, Juan F. García-Reyes, Joachim Franzke

**Affiliations:** † Analytical Chemistry Research Group, Department of Physical and Analytical Chemistry, University of Jaén, Campus Las Lagunillas, Jaén 23071, Spain; ‡ ISAS—Leibniz Institut für Analytische Wissenschaften, Bunsen-Kirchhoff-Str. 11, Dortmund 44139, Germany

## Abstract

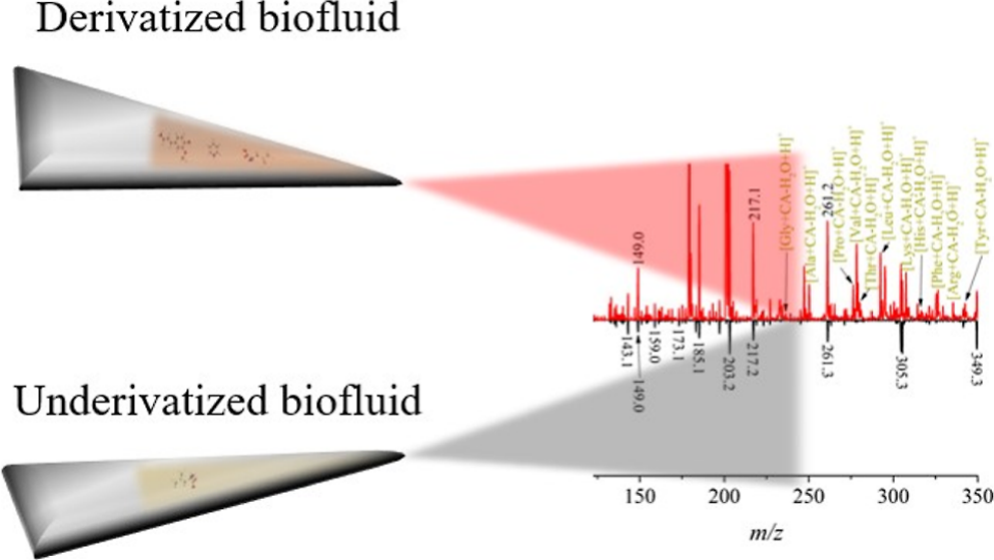

Paper spray mass spectrometry (PS-MS) has evolved into a promising tool for monitoring reactions in thin films and microdroplets, known as reactive PS, alongside its established role in ambient and direct ionization. This study addresses the need for rapid, cost-effective methods to improve analyte identification in biofluids by leveraging reactive PS-MS in clinical chemistry environments. The technique has proven effective in derivatizing target analytes, altering hydrophobicity to enhance elution and ionization efficiency, and refining detection through thin-film reactions on paper, significantly expediting reaction rates by using amino acids (AAs) as model analytes. These molecules are prone to interacting with substrates like paper, impeding elution and detection. Additionally, highly abundant species in biofluids, such as lipids, often suppress AA ionization. This study employs the Schiff base (SB) reaction utilizing aromatic aldehydes for AA derivatization to optimize reaction conditions time, temperature, and catalyst presence and dramatically increasing the conversion ratio (CR) of formed SB. For instance, using leucine as a model AA, the CR surged from 57% at room temperature to 89% at 70 °C, with added pyridine during and after 7.5 min, displaying a 43% CR compared to the bulk reaction. Evaluation of various aromatic aldehydes as derivatization agents highlighted the importance of specific oxygen substituents for achieving higher conversion rates. Furthermore, diverse derivatization agents unveiled unique fragmentation pathways, aiding in-depth annotation of the target analyte. Successfully applied to quantify AAs in human and rat plasma, this reactive PS-MS approach showcases promising potential in efficiently detecting conventionally challenging compounds in PS-MS analysis.

## Introduction

Chemical derivatization stands as a critical resource in enhancing sensitivity, specificity, and overall performance of mass spectrometry (MS) for analyzing challenging compounds^1,2^ Traditionally performed offline, derivatization has undergone innovative adaptation in MS, particularly leveraging the intrinsic properties of electrospray ionization (ESI), resulting in the emergence of “reactions in reduced scale”.^3−5^ This approach involves spray-based ionization coupled with MS, enabling real-time monitoring of reactions in micronano scale droplet reactors, facilitating efficient reactant mixing and interaction.^6,7^ Another strategy involves thin films, enabling faster chemical processes by drop-casting reactant-containing solutions onto surfaces.^8,9^ Reduced-scale reactions in these setups promote rapid solvent evaporation, concentrating reactants due to lower volumes, along with increased surface-to-volume ratios, thereby enhancing reaction kinetics and efficiency.^10^

Within the domain of ambient MS techniques, paper spray (PS)-MS aligns with the reduced-scale reaction paradigm. This research field has fostered the concept of reactive PS, initially serving as a complementary tool to delve into chemical reaction mechanisms within thin films and microdroplets.^11^ Various reactions like the Katrizky reaction,^12^ Haloform reaction,^13^ and nucleophilic substitution reactions,^14^ have been explored within this realm. Reactive PS-MS innovatively leverages these diverse chemical reactions to enhance the detection and quantification of analytes, spanning estrogens,^15^ aldehydes,^16,17^ aryl bromides,^18^ amino acid (AA) neurotransmitters,^19^ among others.

AAs play a vital role in diverse biological processes, serving as the fundamental building blocks of proteins, neurotransmitters, hormones, and various other biomolecules.^20^ They participate in essential processes such as recycling, transamination, and energy production.^21^ AAs are crucial in newborn screening (NBS) analysis, especially for detecting inborn errors of metabolism (IEM).^22^ Early diagnosis of an IEM is critical to prevent premature deaths and mitigate potential long-term developmental impairments in newborns.

The NBS procedure begins with collecting a small amount of an infant’s blood on filter paper cards, forming dried blood spots (DBS). This format is compatible with PS coupled with MS.^23^ PS-MS has emerged as a robust technique for direct sample analysis with minimal or no sample preparation, particularly for detecting compounds in complex matrices like urine, blood, plasma, and tissues.^24−26^ Its quantification and identification capabilities in biofluids have been demonstrated for analytes such as illicit drugs,^27^ therapeutic drugs,^28^ biomarkers,^29^ and biomolecules.^30^ PS-MS has gained popularity as an ambient MS technique due to its cost-effectiveness and user-friendly nature. However, the detection of small polar molecules such as AAs presents challenges in PS-MS analysis. The effectiveness of elution is hindered by various weak interactions such as hydrogen bondings and van der Waals forces between paper fibers and polar moieties within molecules.^24^ Additionally, ionization of compounds at low concentrations might be suppressed by highly abundant species in biofluids and tissues, like lipids.^31,32^ The intricate matrix and substrate interactions present significant hurdles for the PS-MS analysis of AAs.

This study introduces an innovative approach that combines on-paper Schiff base (SB) derivatization with PS-MS for the analysis of AAs. The aim is to improve the sensitivity and selectivity of these molecules during biofluid and tissue analysis using PS-MS, offering a rapid, cost-effective, and scalable solution suitable for high-throughput analysis.^33^ We systematically optimized the reaction parameters for the in situ derivatization of primary amines such as AAs, employing various aromatic aldehydes. Factors such as temperature, reaction time, presence of catalysts, and substituents in the carbonyl derivatization agent’s aromatic ring were methodically evaluated to maximize the conversion ratio (CR). The optimized reaction method was successfully employed to determine AAs in human and rat plasma samples, simulating the analysis of dried biofluid spots commonly used for in-bred IEM analysis.

## Experimental Section

### Chemicals and Materials

Acetonitrile (ACN), methanol (MeOH), and water (LC-MS) were procured from Merck-Sigma (Madrid, Spain). Pyridine and ammonium formate were also obtained from Merck-Sigma (Madrid, Spain). Formic and acetic acids were sourced from Scharlab (Barcelona, Spain). Whatman no. 1 and Whatman 903 Protein Saver Card papers were acquired from Cytiva (Buckinghamshire, UK) through Merck-Sigma (Madrid, Spain).

Analytical standards of leucine (analytical standard) and 4-hydroxy-3-methoxycinnamaldehyde or coniferyl aldehyde (CA) (≥98%) along with 1,3-benzodioxole-5-carboxaldehyde, 3,4-(methylenedioxy)benzaldehyde (piperonal), 4-methoxybenzaldehyde (4-anisaldehyde), 4-hydroxybenzaldehyde, 4-methylbenzaldehyde, 4-chlorobenzaldehyde, and benzaldehyde (≥95%) were purchased from Merck-Sigma (Madrid, Spain).

The AAs analytical standard mixture comprised 2.5 mM l-alanine (Ala), l-arginine (Arg), l-aspartic acid (Asp), l-glutamic acid (Glu), glycine (Gly), l-histidine (His), l-isoleucine (Ile), l-leucine (Leu), l-lysine (Lys), l-methionine (Met), l-phenylalanine (Phe), l-proline (Pro), l-serine (Ser), l-threonine (Thr), l-tyrosine (Tyr), and l-valine (Val) in 0.1 N HCl. l-cystine was included at a concentration of 1.25 mM. The certified reference material TraceCERT 17 isotopically enriched (IE) AAs, each at a concentration of 2.5 mM, included Ala-^13^C_3_,^15^N, Arg-^13^C_6_, Asp-^13^C_4_, Glu-^13^C_5_, Gly-^13^C_2_,^15^N, His-^13^C_6_, Ile-^13^C_6_-^15^N, Leu-^13^C_6_,^15^N, Lys-^13^C_6_, Met-^13^C_5_,^15^N, l-Phe-^13^C_6_, Pro-^13^C_5_, Ser-^13^C_3_,^15^N, Thr-^13^C_4_, Tyr-^13^C_6_, Val-^13^C_5_ and 1.25 mM l-Cystine-_13_C_6_,^15^N_2_. All materials were acquired from Merck-Sigma (Madrid, Spain). The AA analytical standard and the IE AAs were diluted to working concentrations using methanol (MeOH).

The human plasma was purchased from Merck-Sigma (Madrid, Spain). The rat plasma was derived from 300 to 400 μL of blood collected from a C57BL/6 wild-type adult mouse. After the collection, the blood underwent centrifugation at 3000 rpm for 10 min to separate the plasma.

### SB-PS-MS Derivatization

**Figure 1:**
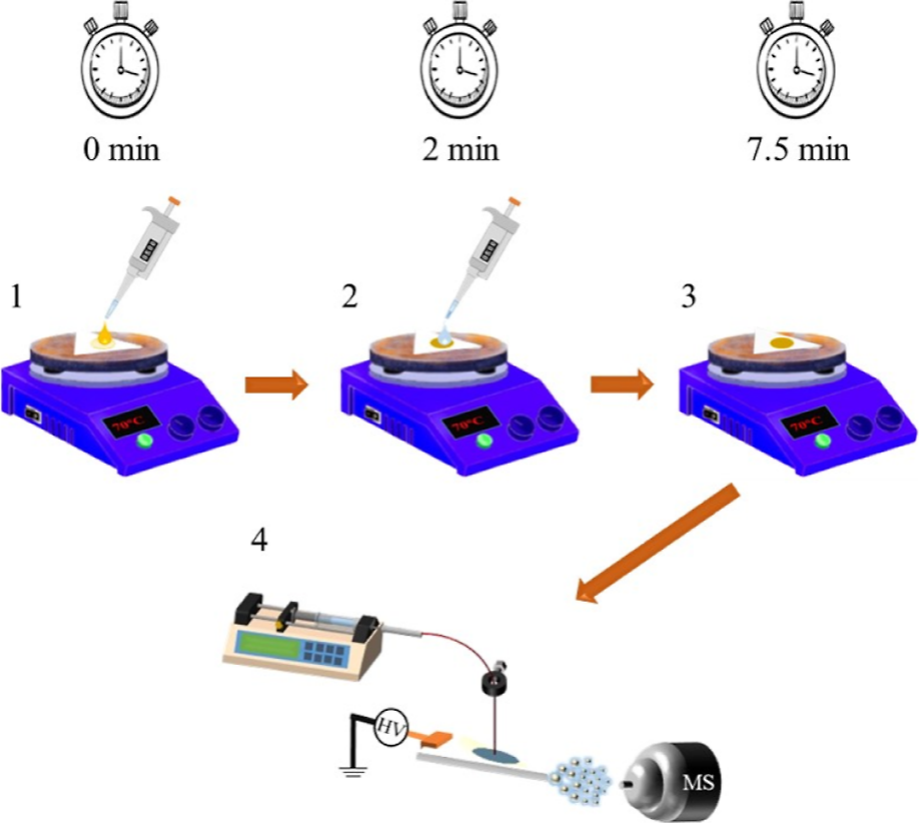
General scheme of the derivatization process on the hot plate at 70 °C with the following steps: 1. addition of the derivatization agent atop the plasma sample, 2. after 2 min, the catalyst is added, 3. the reaction is allowed to proceed for 5.5 min, and 4. subsequently, the paper is positioned in front of the mass spectrometer for PS-MS analysis.

The general derivatization steps are illustrated in Figure 1. For PS analysis, a triangular piece of paper with dimensions of 12 mm (base) × 19 mm (height) was used. Five microliters of the sample (standard or plasma) were deposited on the paper and allowed to dry for 1 h.

The optimization of the derivatization process involved measuring three replicates of the Leu standard deposited on three different paper substrates for subsequent treatment and analysis.

#### Quantification Approaches

- Premix approach:
  - Plasma and IEAAs were mixed before deposition on the paper.
  - Final concentration of targeted IEAA (Gly, Ala, Ser, Pro, Val, Thr, Leu + Ile, Asp, Phe, Arg, and Tyr) in the deposited solution: 62.5 μM.
- On-paper approach:
  - Five microliters of plasma were deposited on the paper and allowed to dry for 1 h.
  - Subsequently, 5 μL of a 62.5 μM IEAA solution were deposited on the dried sample and allowed to dry for an additional 30 min before derivatization.

#### Derivatization Process

1. After drying, the paper with the sample was placed on a hot plate (IKA RCT Standard, Staufen, Germany) at 70 °C for 1 min.
2. Subsequently, 5 μL of the derivatization agent were applied to the paper, initiating a timer.
3. After 2 min, pyridine was deposited on the top of the paper.
4. At 5.5 min postpyridine deposition, the sample was positioned in front of the mass spectrometer for the subsequent addition of the PS solvent for analysis.

### SB-PS-MS Analysis

After the derivatization for PS-MS analysis, 20 μL of MeOH/water (9:1) with 0.1% formic acid was applied to the paper to halt the reaction and moisten the paper. Subsequently, a continuous flow of 30 μL/min of the same solvent was directed to the paper to sustain the signals and prevent complete solvent depletion during analysis. The continuous flow was maintained using a syringe pump connected to a red peek tube (1/16 in. outer diameter × 0.005 in. inner diameter), creating a meniscus with the paper surface.

Two mass spectrometers were employed for the analysis: Thermo Finnigan LTQ linear ion trap mass spectrometer (Thermo Scientific, San José, CA, USA) for SB-PS-MS characterization and TSQ Quantiva triple quadrupole mass spectrometer (Thermo Fisher Scientific, San José, CA, USA) for quantification.

The paper for PS-MS analysis was positioned 10 mm away from the mass spectrometer entrance in both cases by using an in-house assembly with components from Thorlabs (Newton, NJ, USA). For the LTQ, PS was operated at 5000 V, with a capillary temperature of 250 °C, capillary voltage of 20 V, and tube lens voltage of 100 V. The collision-induced dissociation had a nonoptimized fragmentation energy, and 25% normalized collision energy units were used. For the TSQ Quantiva, PS was operated at 4500 V, with an ion-transfer tube temperature of 300 °C. The details of multiple reaction monitoring (MRM) experiments parameters are provided in the Supporting Information (Table S1).

The derivatized AAs were labeled with the AA three-letter code, the derivatization agent [(4-hydroxy-3-methoxycinnamaldehyde or coniferyl aldehyde (CA)], the water loss (–H_2_O), and the adduct ion. The Leu signal is the sum of the Leu and Ile.

### LC-QqQ Analysis

The analysis utilized ultrahigh-performance liquid chromatography (UHPLC)-MS/MS with a Dionex Ultimate 3000 UHPLC system (Thermo Fisher Scientific, Waltham, MA, USA). Chromatography employed a Waters Acquity UHPLC BEH Amide column (1.0 mm × 150 mm, 1.7 μm) with an Acquity guard filter. MS was performed on a TSQ Quantiva instrument in positive ion mode with ESI.

The UHPLC chromatographic details, including the gradient, are specified in the Supporting Information (Table S2). For mass spectrometric analysis, the instrument operated in positive ion mode with ESI as ion source. Relevant MS parameters and MRM experiments are outlined in the Supporting Information (Table S3a,b). Quantification was performed using Skyline software.^34^

The mass spectra obtained from SB-PS-MS, PS-QqQ, and LC-QqQ data were subjected to analysis and manual examination using the XCalibur 3.0 software package developed by Thermo Scientific.

### LC-MS Plasma Samples Preparation

The plasma samples were stored at −20 °C before analysis. The samples were thawed on ice. Ten microliters of plasma were combined with 150 μL of the IEAA mixture, each at a concentration of 10 μM (excluding cystine, not considered for quantification). The Eppendorf containing the mixture was capped, vortexed for 10 s, and stored at −20 °C for 10 min to induce protein precipitation. Subsequently, the samples underwent centrifugation at 13,500*g* for 10 min. The resulting supernatant was carefully transferred to a clean vial and subjected to evaporation under a nitrogen atmosphere for at least 30 min or until complete dryness. The dried sample was reconstituted in 50 μL of phase B (acetonitrile with 0.1% formic acid).^35^

## Results and Discussion

### PS-MS Plasma Analysis

**Figure 2:**
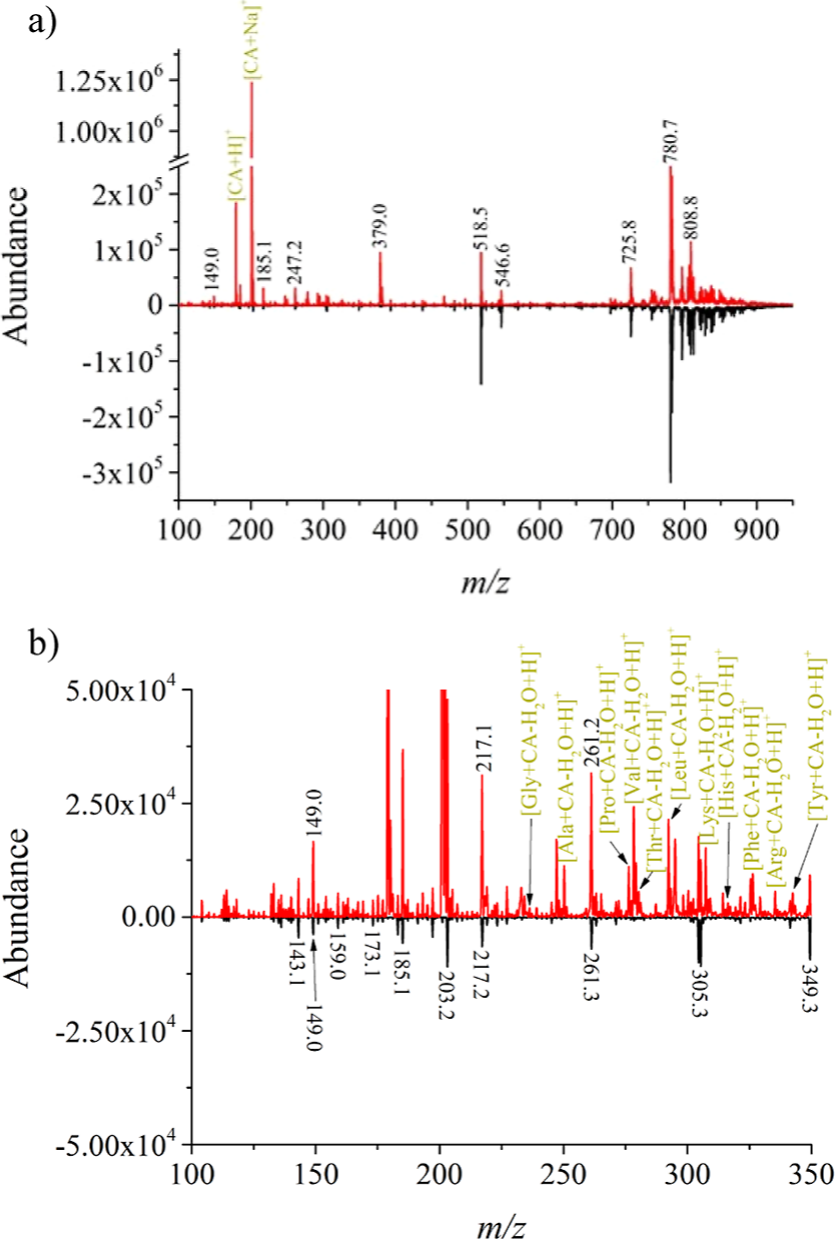
(a) Mass spectra of 5 μL of human plasma analyzed by PS-MS with derivatization (red trace) and without derivatization (black trace), and (b) zoom-in on the *m*/*z* region for AAs detection.

In our initial experiments, we optimized our method using a Thermo LTQ ion trap. We began by analyzing 5 μL of human plasma directly on triangular-cut paper, allowing it to dry for an hour. The PS-MS mass spectrum without derivatization (depicted as the black trace in Figure 2a) was dominated by polar glycerolipids (*m*/*z* > 700), with minimal to no signal for small polar molecules such as AAs, as previously reported.^32^

However, the derivatization process significantly enhanced the signals of small molecules, as indicated by the red trace in Figure 2. With a high concentration of coniferyl aldehyde (CA) (>100 mM), the spectrum featured *m*/*z* 179.1 ([CA + H]^+^) and the sodiated adduct ([CA + Na]^+^) at *m*/*z* 201.1. While lipids were still ionized, a closer examination of the *m*/*z* region where AAs should be detected (Figure 2b) revealed multiple signals for proteinogenic AA, including Gly, Ala, Pro, Val, Thr, Leu, Ile, Lys, His, Phe, Arg, and Tyr.

The AAs were detected because imine formed during the reaction. The imines, having a lower p*K*_a_ (∼7) compared with the average p*K*_a_ of ∼9 in AAs, are still basic enough to form iminium ions through imine protonation during ESI analysis. However, the introduction of hydrophobicity by the CA’s aromatic ring reduced the weak interactions of the AAs with the paper, facilitating elution during PS-MS analysis. Another potential advantage of the hydrophobic nature of the formed SBs is improved ionization, as the concentration of hydrophobic analytes on the droplet’s surface enhances the probability of charging during the ESI process.^36^

### Optimization of the Derivatization Reaction

**Scheme 1:**
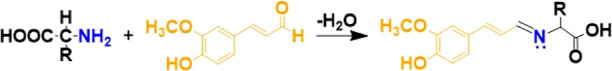
Simple Representation of the SB Reaction between an AA and CA

Leucine was chosen as the representative AA, and CA was the selected model aldehyde for optimizing the SB reaction conditions. Derivatizing AAs with CA results in an *m*/*z* shift of 160 Da. In the case of Leu, this reaction yields a protonated imine detected at *m*/*z* 292.2, signifying the product formed from the interaction between CA and Leu, accompanied by the loss of water, as depicted in Scheme 1. The tandem MS spectrum of *m*/*z* 292.2 (Figure S1a) illustrates the characteristic fragments observed for these SBs. The removal of CO + H_2_O (−46 Da) generates a product ion at *m*/*z* = 246.2. Additionally, a product ion at *m*/*z* 161.1 is noticeable, representing the protonated ion of the dehydrated CA after removal of the AA moiety (Figure S1b). As we will see in the following sections, this specific product ion resulting from the derivatization agent proves to be a suitable choice for quantifying AAs in plasma.

An early observation made during the optimization process was the suboptimal conversion obtained when the reactions were conducted on paper with equimolar quantities of the AA and the derivatization agent. To initiate the optimization process, establishing an appropriate AA/CA ratio was essential since SB reaction kinetics present challenges when dealing with reactions in small volumes.^10^ Furthermore, in reactive DESI, larger quantities of the derivatization agent are necessary when the sample is deposited first and allowed to dry.^37^ Therefore, the initial ratio chosen for optimizing the reaction was 1:50.

In line with previous studies,^10^ the absence of quantification strategies for the reaction products made it necessary to evaluate the reaction’s efficiency by assuming that the precursor amine and product imine have similar ionization efficiencies, and therefore, the ion abundances are directly proportional to the concentrations. This efficiency assessment employed the CR, defined as the ratio of the intensity of the *m*/*z* corresponding to the formed SB to the sum of the intensities of the limiting reactant, the AA, and the SB intensities, as expressed by eq 1
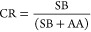
1

**Figure 3:**
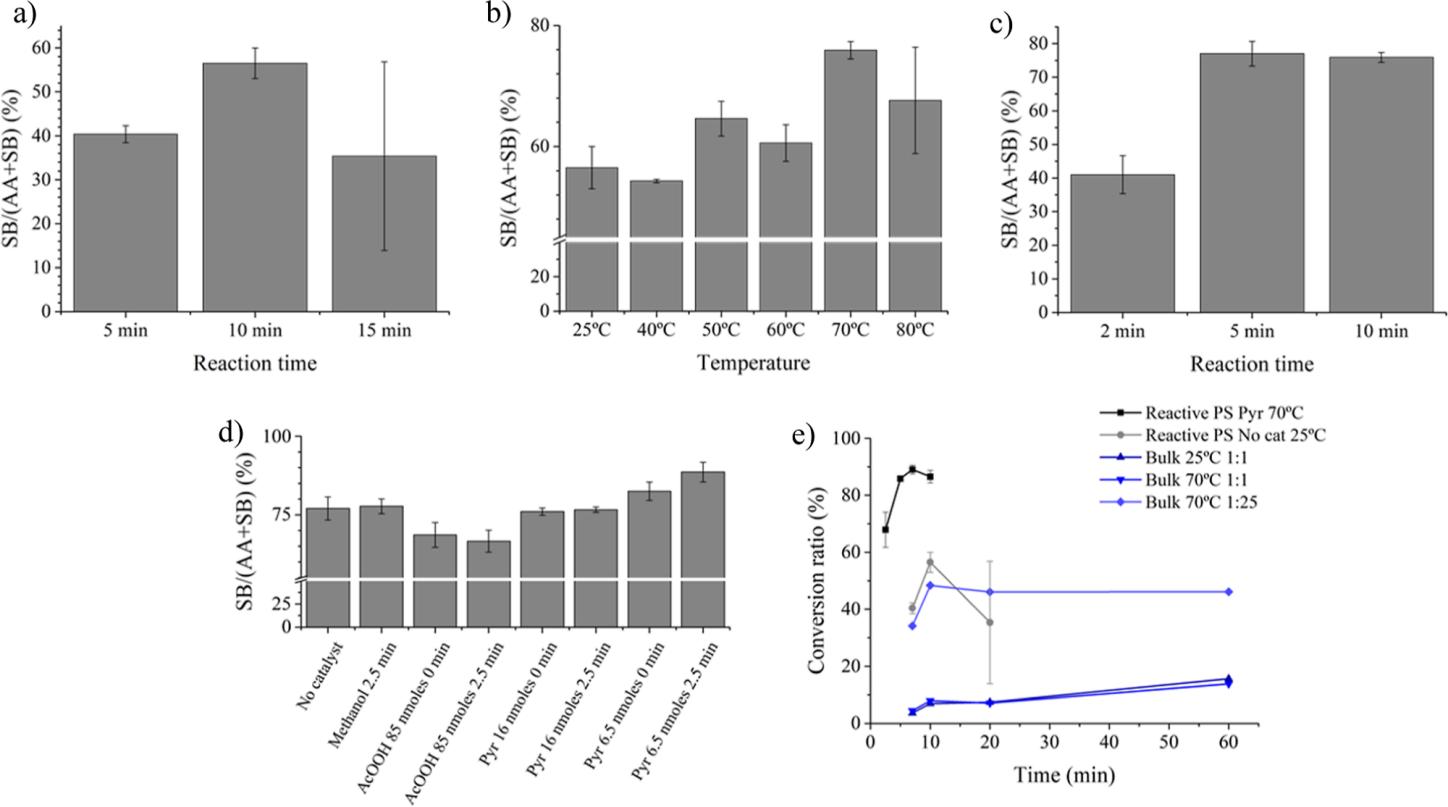
Optimization of reaction conditions using 5 μL of a 500 μM solution of Leu. (a) Influence of reaction time at room temperature; (b) effect of temperature on the CR; (c) reaction time at the optimum temperature (70 °C); (d) effect of the catalyst in the mixture (0 min) or during the reaction (2.5 min); and (e) comparison of on-paper reactions at room temperature and 70 °C, and bulk reaction at different AA-CA ratios and temperatures. AA = amino acid, SB = Schiff Base product of the reaction of the AA and CA.

Initially, our goal was to keep the reaction as straightforward as possible. Nevertheless, as seen in Figure 3a, conducting the derivatization reaction at room temperature yielded a maximum conversion of only 57% after 10 min. Beyond 15 min, the reaction exhibited erratic behavior, with the conversion dropping to 35%. This decline was attributed to potential hydrolysis of the formed SB due to excess water released during the reaction and the slightly acidic environment created by the excess of CA and the methanol used to dissolve CA.

The mechanism of reactive PS was found to rely on solvent evaporation from the paper.^12^ To address the issue of excess water and facilitate evaporation, we opted to raise the reaction temperature while keeping the reaction time at 10 min. Higher temperatures effectively improved the CR, increasing it to 76% at 70 °C (Figure 3b). However, further temperature increases led to a decreased reproducibility. This was to be avoided to preserve the integrity of the paper substrate, particularly beyond 80 °C.

As shown in Figure 3c, higher temperatures accelerated the process, achieving a 77% conversion rate in just 5 min. The mass spectra at different reaction times (Figure S2) indicated a significant increase in the Leu SB ion (*m*/*z* 292.3) after 5 min, stabilizing at the 10-min mark. The type of SB reaction can be influenced by performing the reaction in either an acidic or basic medium.^38^ Different scenarios were explored: for a reaction at 70 °C, 5 μL of methanol was added 2.5 min after adding the CA solution, resulting in no significant change in the final CR (Figure 3d). However, when a weak acid like acetic acid was added either concurrently with CA or 2.5 min after depositing the derivatization agent, reductions of 8 and 10%, respectively, in the CR were observed. In the case of a basic medium, pyridine was employed as a catalyst. The concentration and timing of pyridine application were evaluated, and a CR of 89 ± 3% was achieved when 6.5 nmol of pyridine were deposited 2.5 min after the catalyst deposition. PS-MS measurements were taken 5.5 min after pyridine deposition, resulting in a total reaction time of 7.5 min. Pyridine serves a dual role: it redissolves the reactants, enhancing the interaction of potential stacked compounds on the paper, and facilitates the deprotonation of the imine formed during the Schiff reactions (Scheme S1) easing the dehydration. Typically, this deprotonation role is performed by the target amine (the AAs in our case).

The improvement in derivatization abundances is clearly evident in the mass spectra presented in Figure S3. Starting with an equal quantity of Leu (2.5 nmol) deposited onto the paper, the PS-MS abundances of [Leu + CA-H_2_O + H]^+^ at *m*/*z* 292.3, under optimized derivatization conditions, were nearly twice as high as the abundance of the nonderivatized [Leu + H]^+^ ion at *m*/*z* 132.2.

Following the optimization of the derivatization reaction, the suitability of the AA/CA ratio of 1:50 was examined. A calibration study correlating the product ion signal of Leu derivatization with the amount deposited on the paper, using 125 nmol of CA for derivatization, was conducted. As depicted in Figure S4, the reaction efficiency declined when the ratio was 1:25, while a linear trend was maintained within the ratios of 1:1250 to 1:50.

The optimization of the reactive PS-MS reaction conditions, specifically for the formation of AA SBs, proved to be highly beneficial. It assisted in detecting traditionally challenging target compounds such as AAs during PS-MS analysis, enhanced the sensitivity of polar compounds by increasing hydrophobicity, facilitated analyte desorption from the paper, induced higher ionization efficiencies, and reduced solvent and sample usage for each analysis. This demonstrates the potential of reactive PS-MS to directly analyze compounds such as AAs using PS-MS in complex and scarce biological fluids.

In Figure 3e, the bulk reaction at room temperature, involving an equimolar solution of Leu and CA, reached a maximum CR of 16% after 1 h reaction. Increasing the temperature to 70 °C did not lead to a change in the CR. We observed a 30% increase when the AA/CA ratio was adjusted to 1:25, and the reaction was maintained at 70 °C. This matched the conversion rates achieved during the 10 min derivatization on paper at room temperature and even surpassed the erratic 15 min derivatization analysis. Nonetheless, these numbers still fell short of the optimized reactive PS derivatization, which achieved higher CRs (89%) in shorter durations (7.5 min). The acceleration of the reaction on paper is believed to be favored by the formation of a liquid film, facilitated by the charged microdroplets, or a combination of these factors. This is dependent on the extent of solvent evaporation linked to increased reagent concentrations, pH changes, and enhanced intermolecular interactions.^14^

### Aromatic Aldehydes Substituents and Amino Acids Reactivity

To further optimize the reaction conditions, we examined the impact of aromatic aldehydes on AA analysis. Seven aldehydes CA, piperonal, 4-anisaldehyde, 4-hydroxybenzaldehyde, 4-methylbenzaldehyde, 4-chlorobenzaldehyde, and benzaldehyde were chosen for their known ability of forming stable imines for the selected reaction.^39^ The chemical structures are detailed in Figure S5. In our analyses, a 100 μM solution of a commercial AA mixture (17 AAs) was used, as detailed in the Experimental section. Ala and cystine were excluded of the present evaluation due to interferences at their [M + H]^+^ ions (*m*/*z* 90.1 and 241.2), and Ile and Leu were combined due to undistinguishable signals using the present approach.

**Figure 4:**
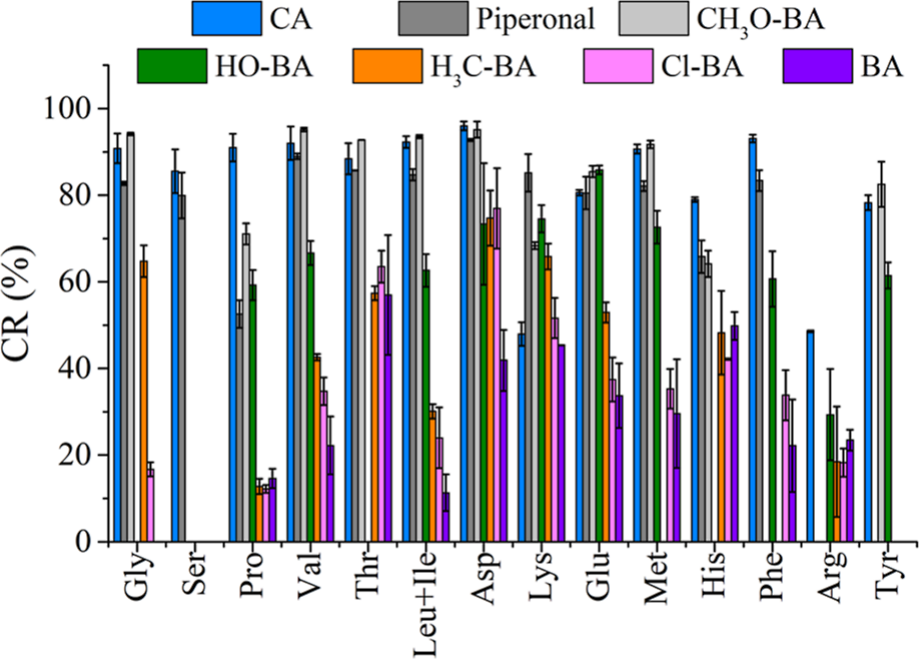
Conversion rates for the commercial mix of AAs derivatized under optimized conditions with each of the seven aromatic aldehydes evaluated: CA (blue), piperonal (dark gray), 4-anisaldehyde or CH3O-BA (light gray), 4-hydroxybenzaldehyde or OH-BA (green), 4-methylbenzaldehyde or H3C-BA (orange), 4-chlorobenzaldehyde or Cl-BA (pink), and benzaldehyde or BA (purple).

Figure 4 displays comprehensive CRs of the different aldehydes. Notably, the nature of the substituents in the aromatic aldehydes played a significant role. Electron-donating moieties, such as methoxy and/or dioxolane groups in CA, 4-anisaldehyde, and piperonal, were crucial for enhanced reactivity. These groups facilitated carbonyl group protonation, promoting efficient imine formation (see Scheme S1, step 1). Although the hydroxyl group in 4-hydroxybenzaldehyde facilitated a certain degree of reaction, the average CR for AAs decreased by 16% compared to CA, except for Lys and Glu. The importance of electron-donating groups was confirmed by 4-methylbenzaldehyde, 4-chlorobenzaldehyde, and plain benzaldehyde as derivatization agents. Weaker donors, electron-attracting groups, and the absence of donors led to lower CRs.

Trends for CR were consistent among AAs when derivatized with CA, piperonal, and 4-anisaldehyde, but four AAs (Pro, His, Lys, and Arg) exhibited distinct behavior. Proline and histidine demonstrated higher CRs with CA (over a 13% difference) compared to those of piperonal and 4-anisaldehyde. Increased steric hindrance in reactants during imine reactions prevented the required iminium intermediate formation.^40^ CA carbonyl group, with lower steric hindrance, likely influenced its superior performance.

Lysine and arginine were the two AAs among the evaluated AAs, exhibiting a significantly lower CR with CA (48 and 49%). Lysine-CA SB had lower CR than piperonal (85%) and 4-anisaldehyde (68%), and for Arg, piperonal, and 4-anisaldehyde, derivatizations were excluded from this comparison due to low derivatization product abundances and interference with the precursor ion at *m*/*z* 293.3, respectively. The more basic amino groups in the ε position for Lys and the guanidinio group for Arg (p*K*_a_ of 10.54 and 12.48, respectively) likely contributed to their distinct behavior with respect to the other AAs. The higher p*K*_a_ of these moieties, coupled with the presence of an α, β unsaturated aldehyde in CA, allows for a potential double nucleophilic addition at 1,2 (imine formation) or 1,4 positions, with a preference for the latter.^39^ The 1,4 additions of Lys and Arg in the case of CA are expected to yield distinct products with *m*/*z* values at 325 and 353, respectively. However, these products were not assessed via tandem MS analysis.

These unique observations require further research, although they are beyond the scope of this work.

### Tandem MS of the Derivatization with CA, Piperonal, and 4-Anisaldehyde

One of the primary objectives of the derivatization process is to enhance detection, but it should also enable accurate quantification. To achieve this, the presence of distinctive diagnostic product ions is crucial for MRM. Using the three aldehydes that demonstrated higher CR (CA, piperonal, and 4-anisaldehyde), we conducted an evaluation and recorded tandem MS spectra of an AA mixture and their isotopically enriched counterparts (Figures S6–S19).

Distinctive patterns emerged in the primary product ions observed across the three derivatization methods employed. First, CA exhibited a dominant product ion at *m*/*z* 161.1, which corresponds to the CA residue minus the carbonyl group. This was observed for most of the formed SB products, with the exceptions being Lys, His, and Arg. Although the product ions related to the derivatization agent might seem inconvenient, the reaction’s specificity, which is limited to primary amines, coupled with the ability to identify the precursor’s *m*/*z* value and narrow isolation windows (±0.4 Da) in the first quadrupole, strengthened the confidence in the identification. This strategy has been previously proposed for quantifying derivatization products.^17,41^

An interesting aspect of the CA derivatization products revolves around the product ion at *m*/*z* 161.1. With the exception of Gly, the signal of this product ion was accompanied by a [M + H]^+^ fragment corresponding to the respective AA. Corroboration of the protonated AA fragment was supported by the analysis of the IEAA counterparts (Figures S6–S19). Fragmentation of SBs formed with CA revealed two distinctive diagnostic product ions akin to peptide bond fragmentation. This led to the formation of a pseudo *b* ion with the CA fragment ion (*m*/*z* 161.1) and a pseudo *y* ion containing the amino group ([M + H]^+^). Additional product ions, such as the loss of water and H_2_O + CO, provided crucial structural annotations and aided in the identification of the target AA.

In contrast, piperonal and 4-anisaldehyde exhibited product ions for the SBs. A loss of 46 Da [–(H_2_O + CO)] was observed in the majority of the tandem MS spectra. However, PS-MS analysis revealed the presence of multiple precursor ions or nonspecific product ions not observed for the isotopically enriched counterparts, complicating the interpretation of ion trap tandem MS spectra.

For additional details on observed product ions and likely losses for the three agents, refer to the Supporting Information.

### Quantification of Amino Acids in Plasma

CA was selected among the three best derivatization agents (CA, piperonal, and 4-anisaldehyde) for AA derivatization in human and rat plasma samples. CA produced higher or equal abundances for most observed SBs from the commercial mixture of AAs, except for Leu-Ile, Lys, and Phe (Figure S20). Once the SB reaction parameters were optimized, quantification was conducted using a triple quadrupole (QqQ) instrument with IEAA standards. Lower dwell times were employed to ensure a higher number of data points for precise quantification.

To determine AA concentrations in plasma, matrix-free external calibration curves were established for 15 AAs using LC-QqQ across a concentration range of 1–50 μM. The calibration curve parameters can be found in Table S4. Plasma samples underwent a 1:5 dilution with water to align with the concentration range of the calibration curve. The average values from the quantification of three replicates of the plasma samples were considered as real values for subsequent assessment of SB-PS-MS quantification capabilities.

SB-PS-MS applicability involved a semiquantification correlating the abundance of the diagnostic product ion selected for each AA SB with the abundance of the IEAA diagnostic fragment, multiplied by the known concentration of the added internal standard. It was assumed that the derivatization efficiency is similar for AA and the enriched counterpart as long as CA is present in excess. The formula for this semiquantification is provided in eq 2
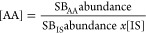
2

Inter- and intraday assays using the premix strategy demonstrated reproducibility with interday relative standard deviations (RSD) under 13% and intraday RSD under 16%, except for Lys, Glu, His, and Met (Tables 1 and 2). The latter four AAs were excluded from further quantification due to the presence of isobars of the precursor for the internal standard *m*/*z*, in the case of Lys-^13^C_6_ and Glu-^13^C_5_ and for His and Met-^13^C^5^–^15^N compromising the accuracy.

**Table 1:** Precision of SB-PS-MS Analysis: Inter-Day Reproducibility was Assessed Over Two Consecutive Days with Five Replicates

AA	inter-day RSD (%)
Gly	0.3
Ala	9.4
Ser	12
Pro	0.1
Val	9.1
Thr	13
Leu + Ile	4.0
Asp	13
Lys	79
Glu	72
Met	90
His	139
Phe	7.9
Arg	10
Tyr	6.4

**Table 2:** Precision of SB-PS-MS Analysis: Intra-Day Analysis was Conducted with Three Triplicates Measured on Three Different Days

AA	intra-day		
	day 1	day 2	day 3
	RSD (%)	RSD (%)	RSD (%)
Gly	4.4	5.0	0.6
Ala	2.7	3.5	1.4
Ser	15	15	1.0
Pro	2.8	3.3	1.6
Val	2.0	1.8	0.9
Thr	14	6.7	1.8
Leu + Ile	3.9	12	0.9
Asp	16	9.5	12
Lys	27	37	12
Glu	59	69	25
Met	16	36	9.5
His	38	38	23
Phe	3.8	1.2	5.9
Arg	13	11	6.9
Tyr	11	2.0	9.1

While SB-PS-MS demonstrated reproducibility, a pivotal question arises: can AAs be accurately quantified in real samples? Using LC-MS concentration results as a reference (Table 3), Gly, Ala, Ser, Pro, Val, Thr, Leu + Ile, Asp, Phe, Arg, and Tyr were quantified employing the premix strategy. The bias of AA concentrations compared to LC references was below 19%, except for Asp, which exhibited low or no intensities, indicating the method’s limit of detection for Asp concentration. Consequently, Asp was not included in the LC quantification. Quantification of the rat plasma sample (Table 4) by SB-PS-MS resulted in a bias under 16% for all evaluated AAs.

**Table 3:** Accuracy of the Premix Strategy Using Whatman 1 Paper for SB-PS-MS Analysis with LC-MS Quantification as the Real Value: Human Plasma^a^

AA	[AA]_human plasma analysis__LC-MS__(μM)_	[AA]_human premix Whatman__1 PS-MS__(μM)_	bias premix_Whatman 1_–LC (%)
Gly	156	140	10
Ala	153	183	19
Ser	90.1	92.5	11
Pro	89.5	102	13
Val	93.3	102	10
Thr	93.1	97.9	5
Leu + Ile	57.2	61.5	8
Asp		21.2	
Phe	35.3	34	12
Arg	152	131	14
Tyr	43.7	47.4	9

a The [AA]_human premix Whatman 1 PS-MS (μM)_ represents the average result from three replicates. For each replicate, the bias was calculated, and the results are presented as bias premix_Whatman 1_–LC (%), which is the average of the bias calculations from the three replicates.

**Table 4:** Accuracy of the Premix Strategy Using Whatman 1 Paper for SB-PS-MS Analysis with LC-MS Quantification as the Real Value: Rat Plasma^a^

AA	[AA]_rat plasma analysis__LC-MS__(μM)_	[AA]_rat premix Whatman__1 PS-MS__(μM)_	bias premix_Whatman 1_–LC (%)
Gly	110	122	11
Ala	231	229	1
Ser	107	121	13
Pro	61.6	71.8	16
Val	78.3	80.6	3
Thr	130	124	4
Leu + Ile	63.2	66.4	5
Asp	36.2	40.2	11
Phe	41.9	42.4	1
Arg	153	144	6
Tyr	40.7	45.6	12

a The [AA]_rat premix Whatman 1 PS-MS (μM)_ represents the average result from three replicates. For each replicate, the bias was calculated, and the results are presented as bias premix_Whatman 1_–LC (%), which is the average of the bias calculations from the three replicates.

The premix strategy was employed to assess the feasibility of quantification when human plasma is deposited on Whatman 903 paper, commonly used for DBS. While the thickness of this paper enhances blood absorption and storage, direct analysis via SB-PS-MS yielded a higher bias (>30%) for most AAs, except for Gly, Val, Leu + Ile, and Phe. Additionally, extremely high concentrations of Ser and Thr were obtained, suggesting interference or artifacts altering quantification. While Whatman 903 Protein Saver Cards are preferred for DBS analysis and storage, Whatman 1 filter paper could serve as an alternative for short-term storage and subsequent analysis of small molecules.^42^ The low-volume storage capabilities of the Whatman 1 filter paper are advantageous for SB-PS-MS.

As samples are routinely collected and stored in medical facilities, we explored the feasibility of adding the isotopically enriched standard mixture after drying the sample on paper. This on-paper approach aims to simplify sample collection compared to the premix strategy, where the sample must be mixed before deposition on the paper card. However, as shown in Table S5, quantification with SB-PS-MS revealed a deficient excess for quantified AAs, except for Gly (10%), Phe (20%), and Tyr (15%), compared to LC-MS analysis. The abundances for the IEAA were lower compared with the premix strategy while maintaining similar levels for plasma AAs. The diffusion of the 5 μL solution containing 62.5 μM of each IEAA was hindered by the dried plasma, limiting even distribution on the paper surface for both AAs and IEAAs, a limitation not present in the premix strategy. The overestimation of concentrations rules out the current on-paper approach for direct quantification.

## Conclusions

The SB-PS-MS method introduced in this study not only promises enhanced detection capabilities but also reduces costs and improves the analytical efficiency for AAs analysis. By reducing the derivatization time to just 7.5 min, the sample treatment optimizes the overall analytical process, offering a method that is both simple and rapid. The proposed reactive PS strategy, employing aromatic carbonyls, significantly boosts the abundances of AA-aldehyde SB products, addressing the challenges of low signal detection observed in biofluids during PS-MS analysis. This streamlined approach accommodates less complex instrumentation, making it accessible to laboratories with budget constraints while eliminating extraction steps, thereby saving time, consumables, and sample quantities.

While derivatization at room temperature with an aromatic aldehyde (CA) allowed for a CR high enough for the sensitive detection of AA SBs by PS-MS, our exploration of optimized reaction conditions underscores the critical role of the reaction conditions on maximizing the CR. The temperature aids in reducing SB hydrolysis, the catalyst boosts the CR, and the appropriate derivatization agent facilitates rapid, efficient, and sensitive reactions. Aromatic aldehydes with electron-donor substituents, particularly those containing oxygen in a basic environment, play a pivotal role in amplifying the formation of SB products, contributing to increased sensitivity, and introducing diverse product ions during tandem MS analysis. These ions, linked to distinctive labile bonds within the SB product, offer insights into the structural characteristics of the analyzed compounds, which is particularly promising for challenging isomers. This multifaceted approach positions reactive PS-MS as a promising avenue for comprehensive structural analysis of hard to separate/differentiate compounds.

Looking ahead, this work highlights the potential of reactive PS-MS in identifying traditionally challenging compounds. Future research should explore the potential of leveraging SB and other reactions to expand detection capacity and facilitate structural identification based on product ions associated with the reaction product and the analyte. The proof-of-concept presented here for AAs warrants further investigation into strategies enabling semiquantification of AAs in NBS for reduced blood volumes (<10 μL) in a high-throughput manner.

## Supporting Information Available

Tandem MS mass spectrum of Leu-SB and structure of *m*/*z* 161; evolution of the mass spectra for three different reaction times of Leu and CA at 70 °C; scheme of the SB reaction; comparison of three reaction scenarios; evaluation of the impact of the ratio between the derivatization agent and AA; structures of various aromatic aldehydes; tandem MS of 15 AAs and their IEAA counterparts (Gly, Ala, Ser, Pro, Val, Thr, Leu/Ile, Asp, Lys, Glu, Met, His, Phe, Arg, Tyr) when derivatized with CA, piperonal, and 4-anisaldehyde; comparison of the abundances with the three selected aromatic aldehydes (CA, piperonal, 4-anisaldehyde); MRM parameters for PS-MS analysis; chromatographic conditions used; LC-QqQ parameters for the ion source and MRM analysis; figures of merit for the LC-QqQ calibrations; comparison of the premix strategy using Whatman 903 and on-paper strategy using Whatman 1 as a paper substrate for PS-MS analysis (PDF)
