# Supplementary material for: Enhanced Compound Analysis Using Reactive Paper Spray Mass Spectrometry: Leveraging Schiff Base Reaction for Amino Acid Detection
Source: Anal Chem. 2024 Mar 20;96(13):5289–97. doi: 10.1021/acs.analchem.4c00215 (PMC10993198; doi:10.1021/acs.analchem.4c00215)
Supplement: Supplementary file 1 — ac4c00215_si_001.pdf [file ac4c00215_si_001.pdf]

Supporting information

Enhanced Compound Analysis Using Reactive Paper Spray Mass Spectrometry: Leveraging Schiff Base Reaction for Amino Acid Detection

Marcos Bouza*[1], Daniel Foest[2], Sebastian Brandt[2], Juan F. García-Reyes[1] and Joachim Franzke[2]

[1]Analytical Chemistry Research Group, Department of Physical and Analytical Chemistry, University of Jaén, Campus Las Lagunillas, 23071 Jaén, Spain.

[2]ISAS—Leibniz Institut für Analytische Wissenschaften, Bunsen-Kirchhoff-Str. 11, 44139 Dortmund, Germany.

[*]**Corresponding authors:** Marcos Bouza, email: mbouza@ujaen.es, phone: +34 953 21 2758.

Table of Contents

Figure S1..S3

Figure S2..S4

Scheme S1..S5

Figure S3..S6

Figure S4..S7

Figure S5..S8

Figure S6..S9

Figure S7..S10

Figure S8..S11

Figure S9..S12

Figure S10..S13

Figure S11..S14

Figure S12..S15

Figure S13..S16

Figure S14..S17

Figure S15..S18

Figure S16..S19

Figure S17..S20

Figure S18..S21

Figure S19..S22

Figure S20..S23

Table S1..S24

Table S2..S25

Table S3..S26

Table S4..S27

Table S5..S28

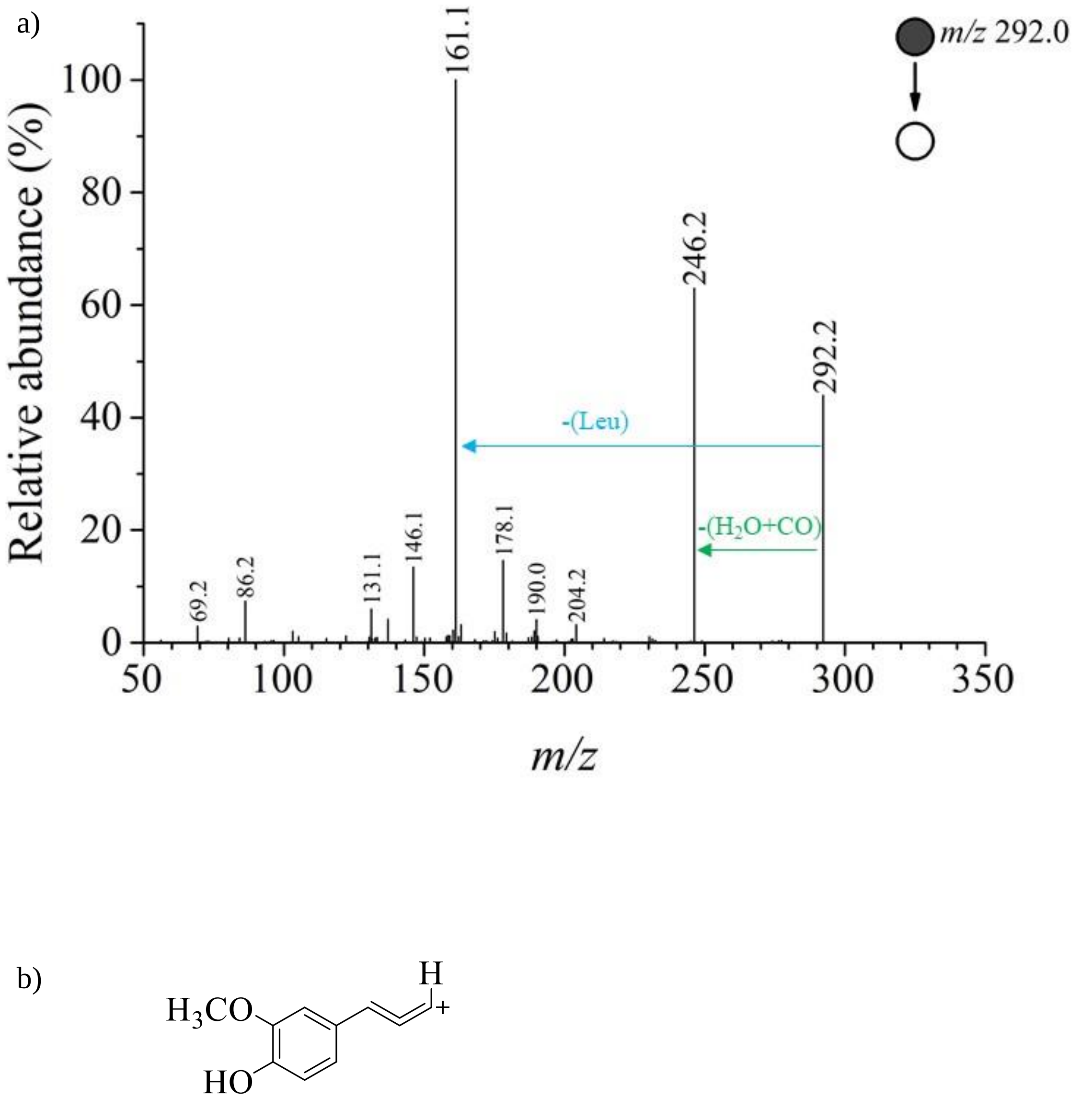

Figure S1. a) Tandem MS mass spectrum at *m/z* 292.0 corresponding to [Leu+CA-H_2O+H]$^+$ and (b) potential structure of coniferyl aldehyde (CA) fragment ion observed at *m/z* 161.1, resembling the protonation and subsequent dehydration of CA.

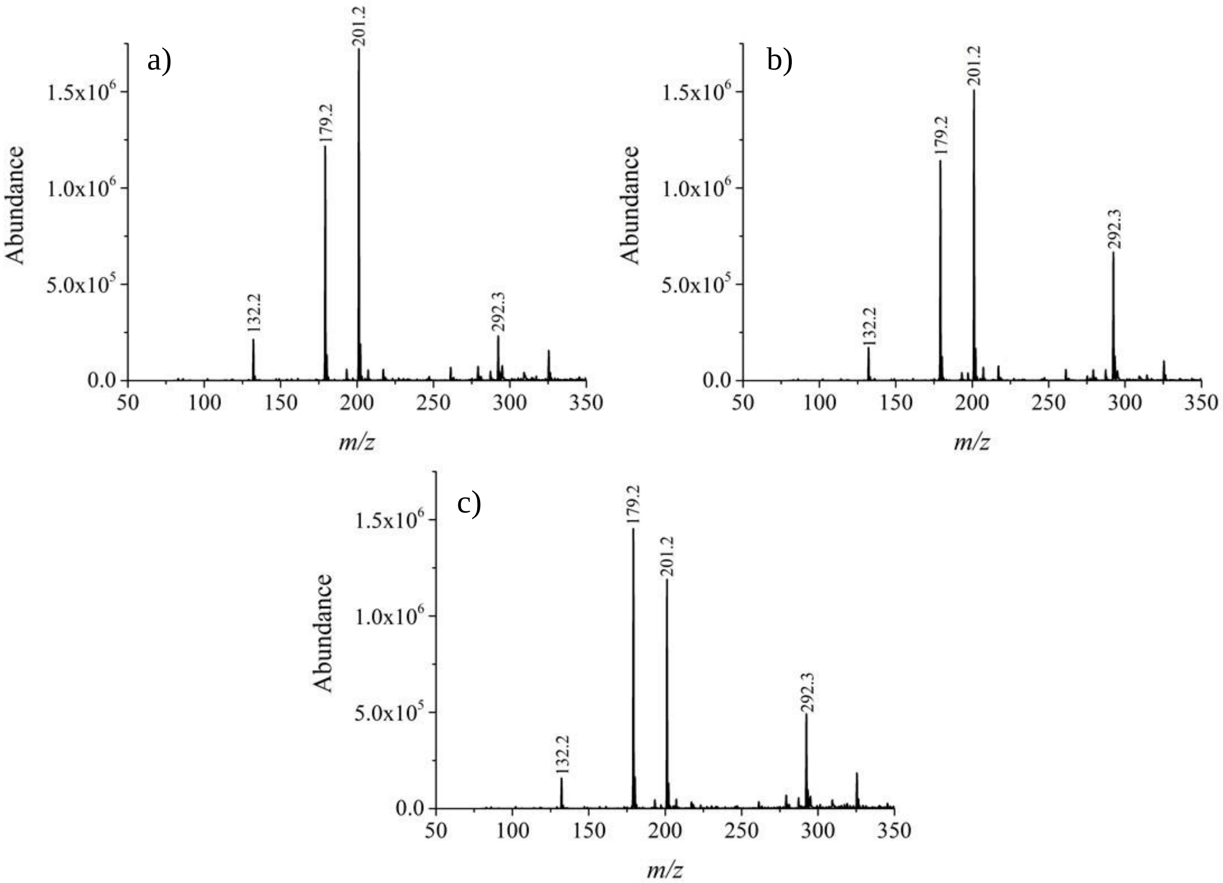

Figure S2. Mass spectra illustrate the Schiff Base (SB) reaction between CA (*m/z* 179.2 $[M+H]^+$ and *m/z* 201.2 $[M+Na]^+$) and Leu (*m/z* 132.2), resulting in [Leu+CA-H2O+H]+ (*m/z* 292.3). The reactions were conducted using reactive PS-MS at 70 ºC and detected after a) 2 min of reaction, b) 5 min of reaction, and c) 10 min of reaction.

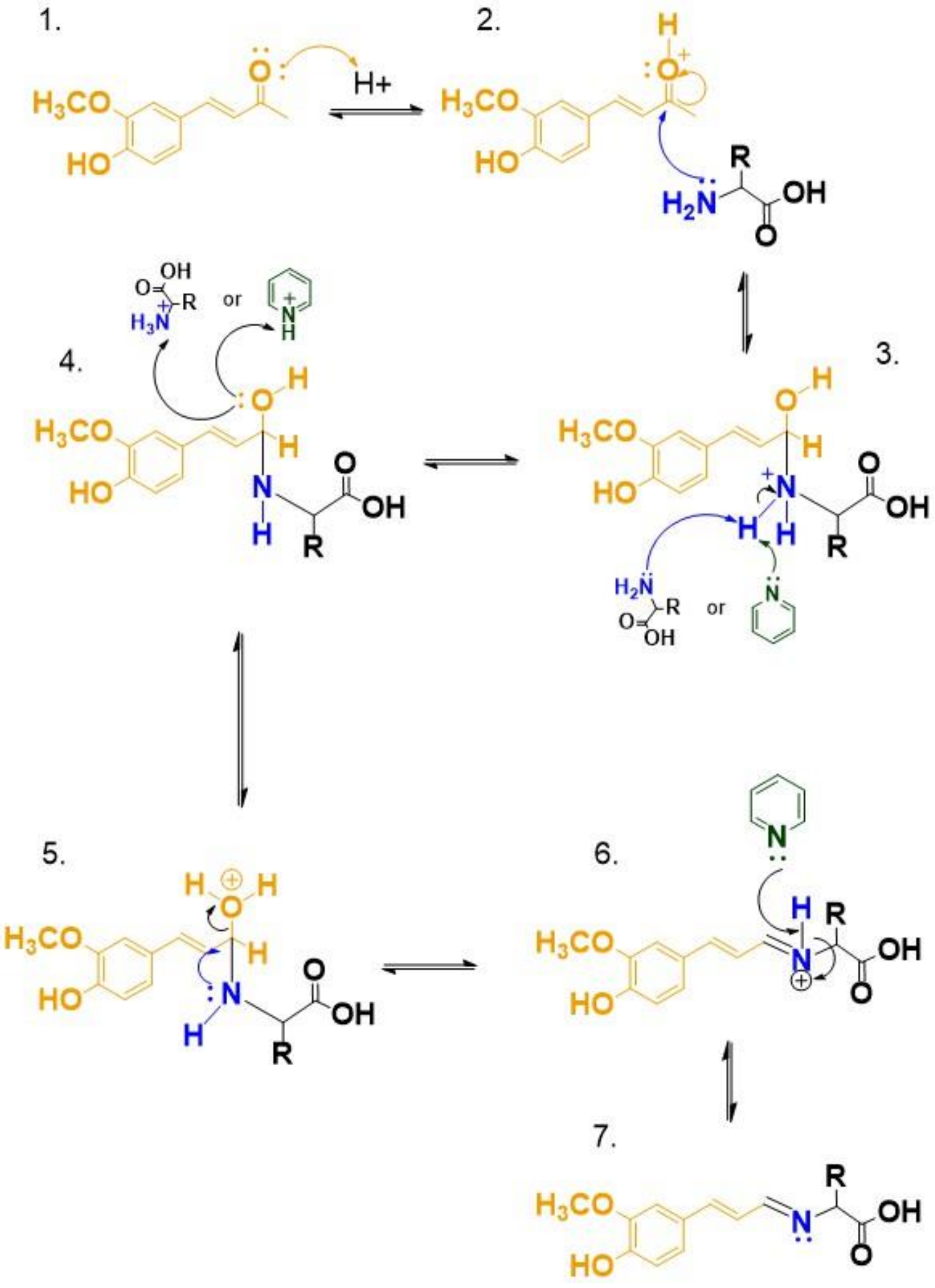

Scheme S1. Proposed mechanism for optimized SB reaction:

1. Protonation of the carbonyl group.
2. Addition of the amine to the protonated carbonyl, followed by charge relocation to form the ammonium.
3. Amino acids (AAs) or later addition of pyridine, acting as bases, to deprotonate the ammonium.
4. Protonation of the oxygen by the acidic AA or pyridine.
5. Water elimination.
6. Deprotonation of the iminium salt to form the final imine (7).

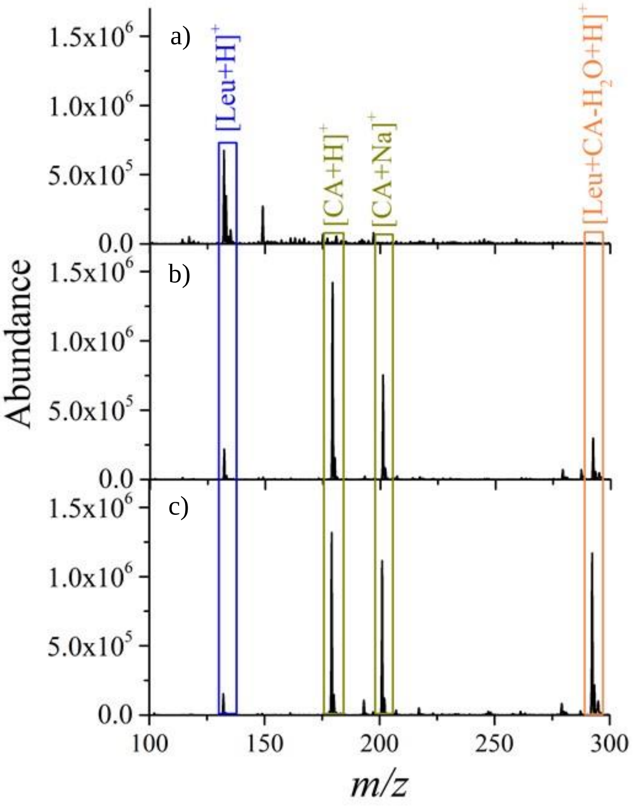

Figure S3. PS-MS mass spectrum of 2.5 nmol of Leu deposited on the paper. a) Non-derivatized, b) 10 min derivatization with 125 nmol of CA at room temperature, and c) 7.5 min derivatization with 125 nmol of CA and 6.5 nmol of pyridine added 2.5 min after the derivatization agent.

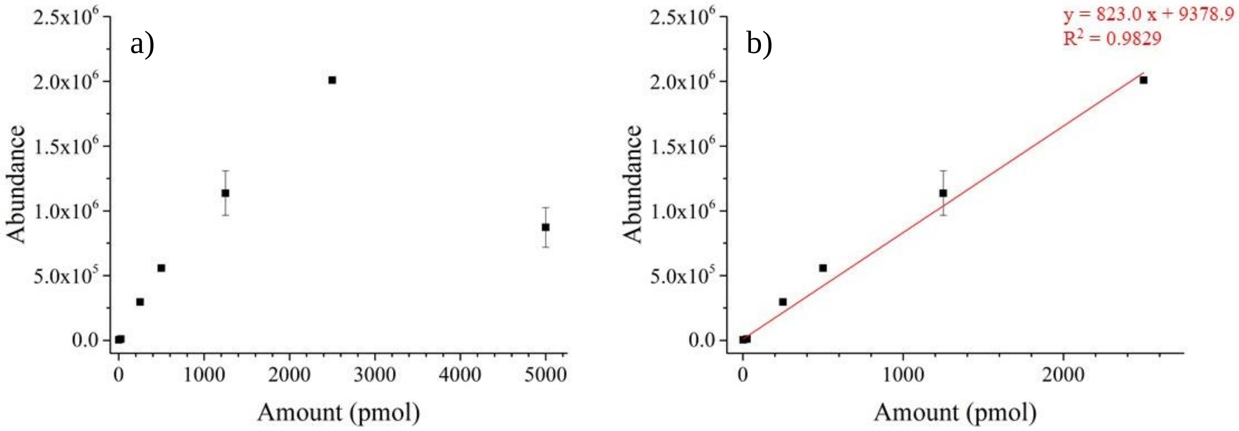

Figure S4. Evaluation of the impact of the ratio between the derivatization agent, CA (maintained at a constant amount of 125 nmol), and the AA, Leu, with varying amounts on the paper from 2.5 pmol to 5000 pmol at 70 ºC. a) Evolution of the abundances of the Leu SB product ion at *m/z* 161 as the amount of Leu increased. b) Calibration curve depicting the region where the Leu SB fragment ion (*m/z* 161) showed a linear relationship with Leu amounts ranging from 2.5 pmol to 2500 pmol.

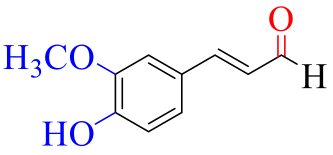

Coniferyl Aldehyde

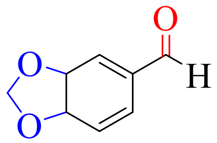

Piperonal

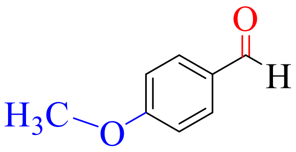

4-anisaldehyde

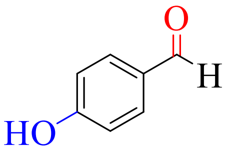

4-hydroxy-
benzaldehyde

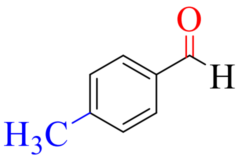

4-methyl-
benzaldehyde

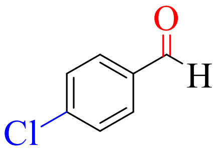

4-chloro-
benzaldehyde

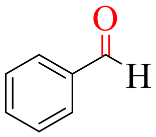

Benzaldehyde

Figure S5. Molecular structures of various aromatic aldehydes assessed for the SB reaction with AAs.

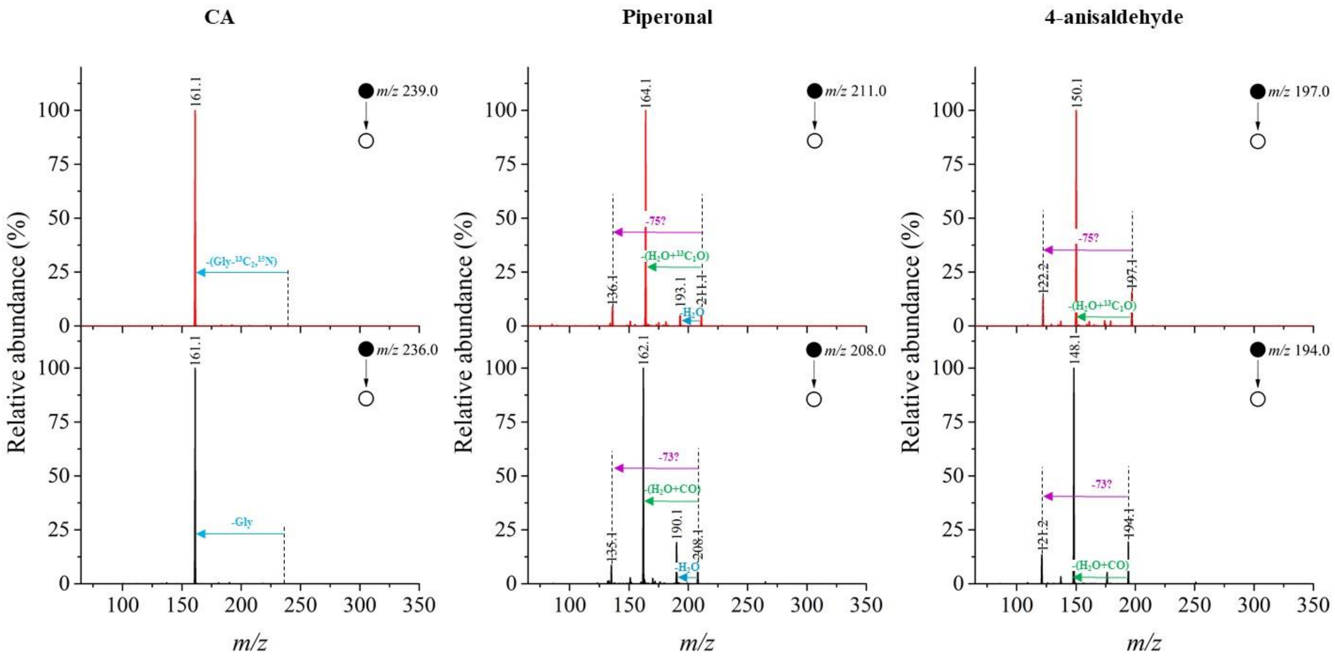

Figure S6. Tandem MS spectra obtained with the Thermo Finnigan LTQ linear ion trap for Gly (black trace) and Gly-$^{13}C_2$,^{15}N (red trace) after derivatization with cinnamaldehyde (CA), piperonal, and 4-anisaldehyde.

Fragmentation of Gly:

<u>CA:</u> the fragmentation of the Gly and CA SB results in the detection of the product ion at *m/z* 161.1, corresponding to the CA fragment of the SB ($[CA\text{-}H_2O+H]^+$), supported by confirmation from the IE standard. Its fragmentation characteristics resemble those of a *b* ion.

<u>Piperonal:</u> when using piperonal as the derivatization agent, Gly exhibits a fragment at *m/z* 190.1 due to water loss, confirmed by *m/z* 193.1 for the IE standard. Furthermore, Gly SB experiences a loss of H_2O+CO, corresponding to *m/z* 162.1. The IE Gly presents a product ion at *m/z* 164.1, indicating the carboxylic CO's ^{13}C presence, resulting in an IE Gly loss of 47 Da instead of 46 Da. Lastly, a product ion at *m/z* 135.1, corresponding to a loss of 73 Da, was detected. The IE standard displayed a fragment ion retaining one of the enriched atoms, resulting in a loss of 75 Da. Both trends suggest some level of atom scrambling involving the imine, necessitating high-resolution MS data accompanied by IR analysis to identify potential intermediates.

<u>4-anisaldehyde:</u> Similar trends to piperonal were observed for 4-anisaldehyde, indicating a loss of H_2O+CO and a fragment ion corresponding to the loss of 73 Da for Gly, and 75 Da for Gly-$^{13}C_2$, ^{15}N.

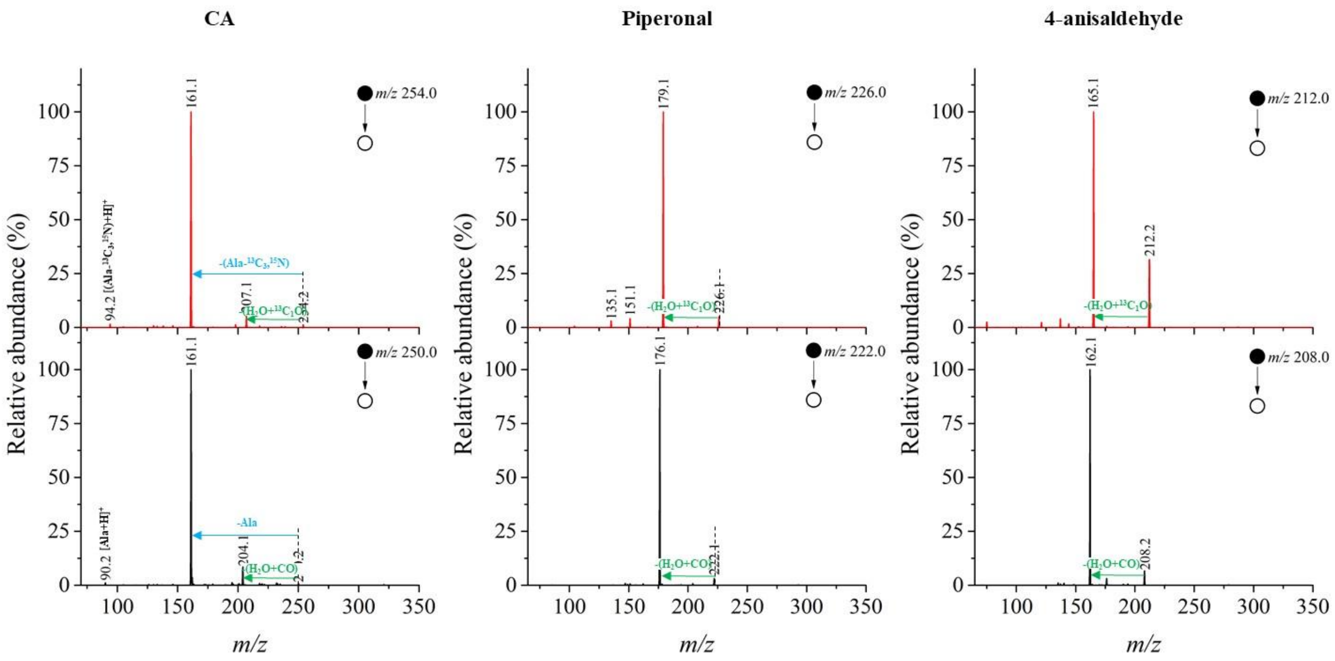

Figure S7. Tandem MS spectra obtained with the Thermo Finnigan LTQ linear ion trap for Ala (black trace) and Ala-$^{13}C_3$,^{15}N (red trace) after derivatization with CA, piperonal, and 4-anisaldehyde. Additionally, the protonated fragment of Ala (*m/z* 90.2) was observed when the molecules was fragmented using 25 NCE; the product ion was corroborated by the IE Ala fragmentation.

Fragmentation of Ala:

<u>CA:</u> In the case of Ala, the tandem MS spectrum revealed the loss of H_2O+CO and a pseudo *b* ion, indicating the loss of Ala (*m/z* 161.1), and the pseudo y ion as $[Ala+H]^+$. The (4-hydroxy-3-methoxyphenyl)prop-2-iminium undergoes fragmentation at the imine double bond via an α-cleavage, producing two structures akin to the fragmentation of the peptide bond.

<u>Piperonal and 4-anisaldehyde:</u> both agents induced SB formation that fragmented by the loss of 46 Da (H_2O+CO).

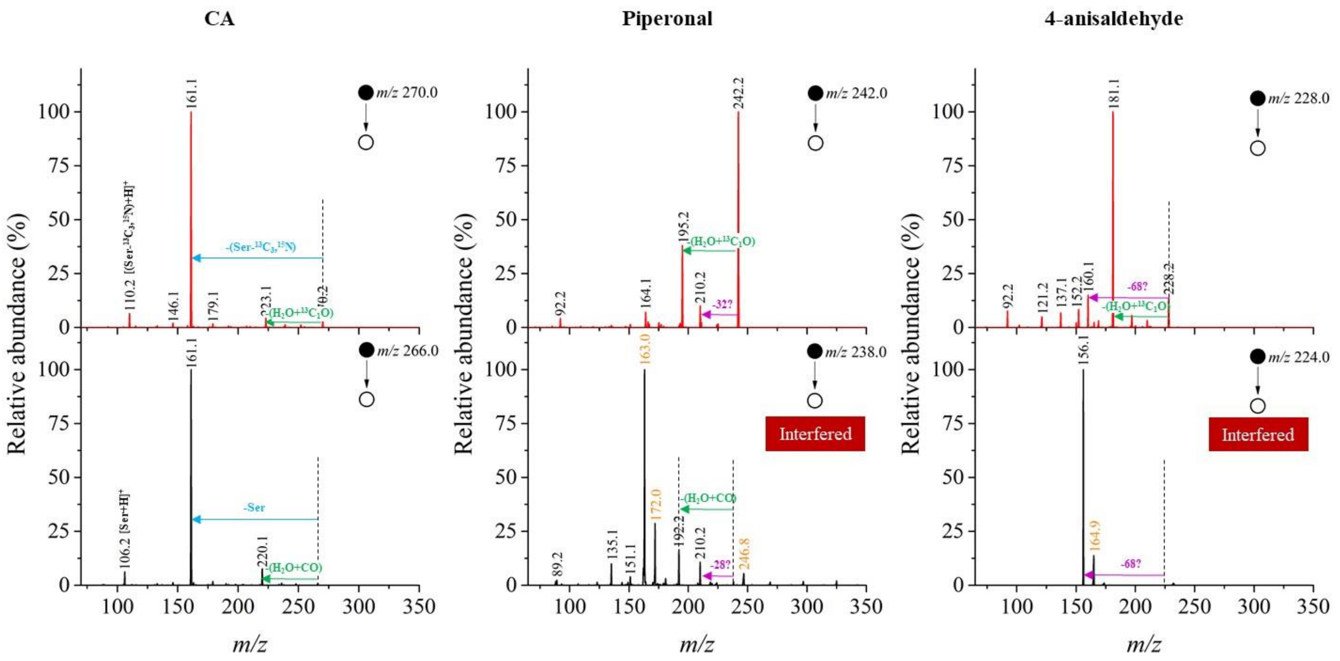

Figure S8. Tandem MS spectra obtained with the Thermo Finnigan LTQ linear ion trap for Ser (black trace) and Ser-$^{13}C_3$,^{15}N (red trace) after derivatization with CA, piperonal and 4-anisaldehyde.

Fragmentation of Ser:

<u>CA:</u> in the case of Ser, the tandem MS spectrum exhibited the loss of H_2O+CO (*m/z* 220.1) and the pseudo *b* ion (*m/z* 161.1) corresponding to the loss of Ser, along with the detection of the pseudo *y* ion $[Ser+H]^+$ (*m/z* 106.2) as a product ion.

<u>Piperonal:</u> the tandem MS mass spectrum of $[(Ser+piperonal)-H_2O+H]^+$ was interfered, showing a doubly charged ion at *m/z* 246.8, even within a 1.5 Da isolation window. Additionally, two fragment ions at *m/z* 163.0 and 172.0, not observed in the IE counterpart, complicated interpretation by the presence of more than one precursor ion. However, the loss of H_2O+CO (-46 Da) was still observable.

The ion observed at *m/z* 210.2 posed another potential challenge. It was observed for both Ser and Ser-$^{13}C_3$, ^{15}N. The slight difference in loss (4 Da) indicated the AA was not fragmented.

<u>4-anisaldehyde:</u> the tandem MS mass spectrum of $[(Ser+4\text{-anisaldehyde})-H_2O+H]^+$ was also interfered. While the IE standard of Ser did not show interference, the dominant product ion was the loss of H_2O+CO detected at *m/z* 181.1. Both spectra displayed a loss of 68 Da, where the 4 Da shift from *m/z* 156.1 for Ser to *m/z* 160.1 for Ser-$^{13}C_3$, ^{15}N indicated a fragment containing the entire AA structure.

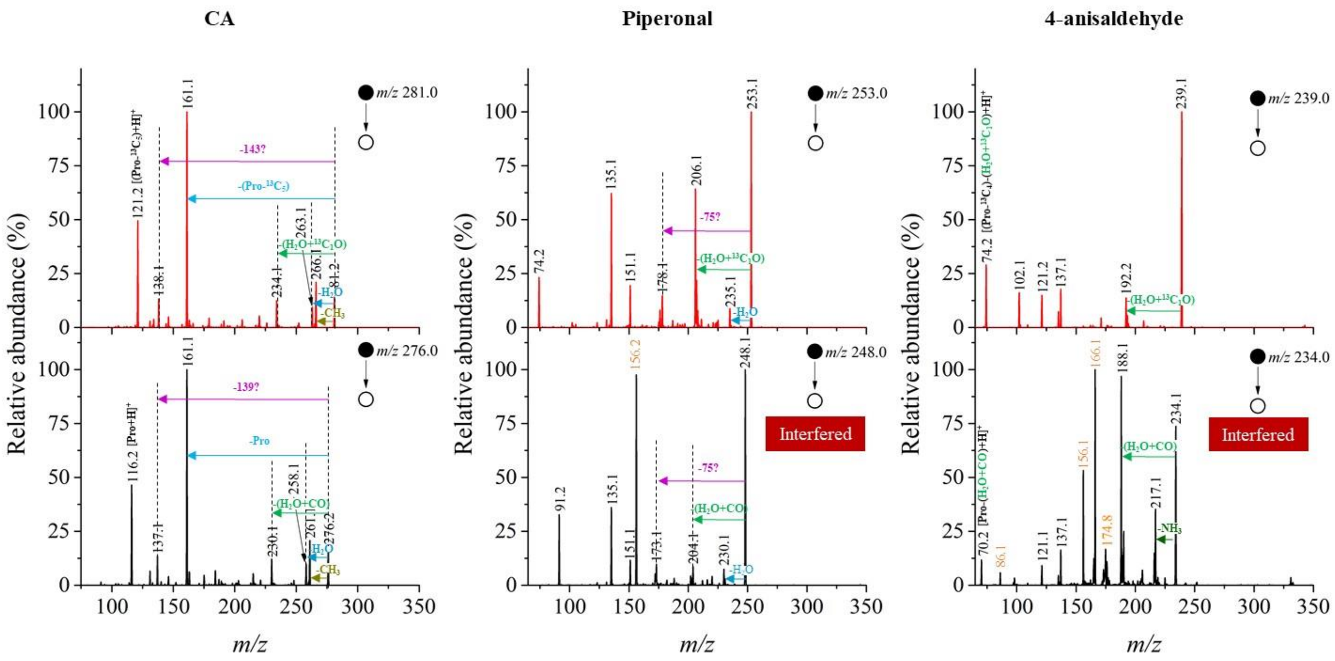

Figure S9. Tandem MS spectra obtained with the Thermo Finnigan LTQ linear ion trap for Pro (black trace) and Pro-$^{13}C_5$ (red trace) after derivatization with CA, piperonal and 4-anisaldehyde.

Fragmentation of Pro:

<u>CA:</u> in the Pro SB, we observed product ions corresponding to the losses of CH_3 (*m/z* 261.1), H_2O (*m/z* 258.1), H_2O+CO (*m/z* 230.1), pseudo *b* ion or loss of Pro (*m/z* 161.1), along with the pseudo *y* ion at *m/z* 116.2 corresponding to $[Pro+H]^+$ residue. However, due to current resolution limitations in our ion trap and low abundances hindering informative MS^n analysis, we could not assign plausible structures for the neutral losses of 139 Da for Pro and 143 Da for Pro-$^{13}C_5$.

<u>Piperonal:</u> the tandem MS mass spectrum of $[(Pro+piperonal)-H_2O+H]^+$ was interfered, showing a unique product ion (not observed in the IE counterpart) at *m/z* 156.2. Nevertheless, several product ions were observed for the SB: water loss at *m/z* 230.1 and H_2O+CO loss at *m/z* 204.1. The neutral loss of 75 Da remained uncharacterized. Additionally, two major product ions at *m/z* 151.1 and 135.1 were observed, indicating the possible product ion from the derivatizating agent at *m/z* 151.1, potentially due to recombination or presence during analysis, as these product ions did not display isotopic enrichment.

<u>4-anisaldehyde:</u> the tandem MS mass spectrum of $[(Pro+4\text{-anisaldehyde})-H_2O+H]^+$ was interfered, showing a doubly charged ion at *m/z* 174.8 and two fragment ions at *m/z* 166.1 and 156.1, not observed in the IE counterpart, complicating interpretation. However, the loss of H_2O+CO (*m/z* 188.1) and the product ion corresponding to the AA and the loss of H_2O+CO (*m/z* 70.2) were also detected.

Similar to piperonal, 4-anisaldehyde SB produced *m/z* 137 and *m/z* 121.1, likely originating from the aldehyde, as there were no signals of isotopic enrichment in the product ion.

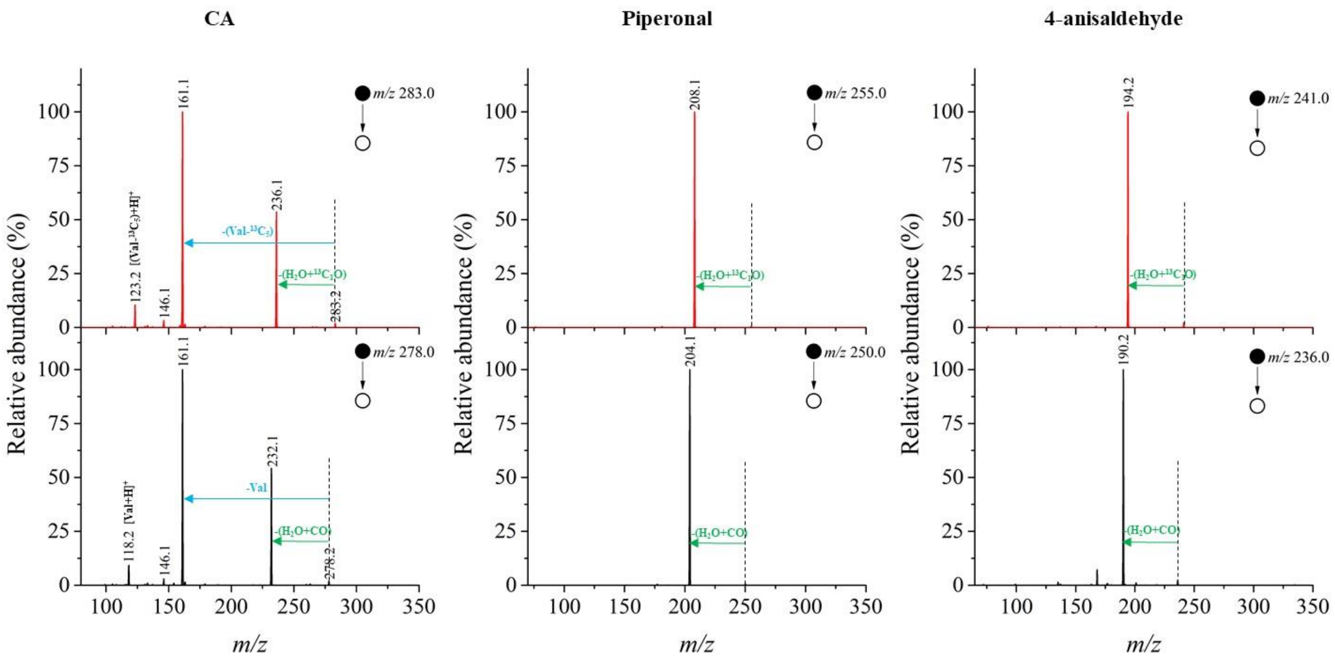

Figure S10. Tandem MS spectra obtained with the Thermo Finnigan LTQ linear ion trap for Val (black trace) and Val-$^{13}C_5$ (red trace) after derivatization with CA, piperonal and 4-anisaldehyde.

Fragmentation of Val:

<u>CA:</u> the tandem MS spectrum displayed the loss of H_2O+CO and the pseudo *b* ion corresponding to the loss of Val (*m/z* 161.1), along with the pseudo *y* ion, $[Val+H]^+$, at *m/z* 118.2.

<u>Piperonal and 4-anisaldehyde:</u> both led to a SB reaction that fragmented through the loss of 46 Da (H_2O+CO).

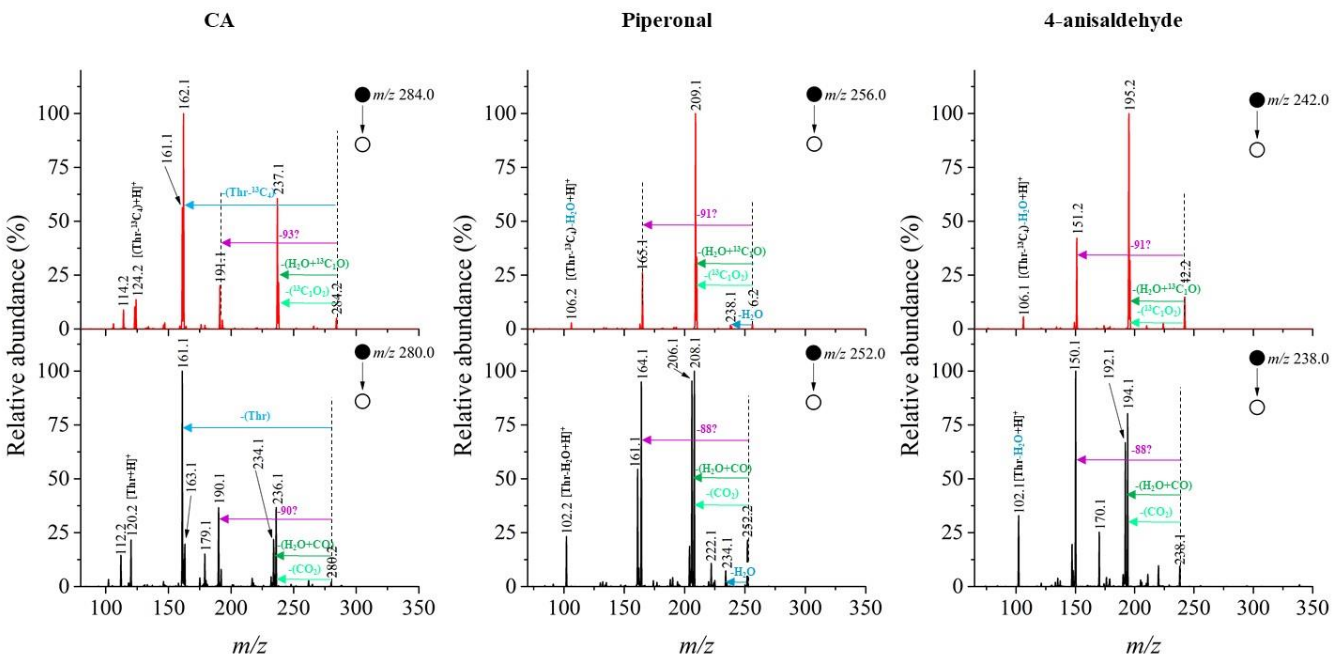

Figure S11. Tandem MS spectra obtained with the Thermo Finnigan LTQ linear ion trap for Thr (black trace) and Thr-$^{13}C_4$ (red trace) after derivatization with CA, piperonal and 4-anisaldehyde.

Fragmentation of Thr:

In all three cases, the Thr SB revealed the loss of H_2O+CO (-46 Da), consistent with the IE AA analysis. Additionally, a loss of -44 Da was annotated and attributed to CO_2. However, the specific fragmentation mechanisms remain unclear, necessitating further investigation with higher-resolution MS.

<u>CA:</u> the tandem MS spectrum displayed the pseudo *b* ion corresponding to the loss of Thr (*m/z* 161.1), and the pseudo *y* ion, $[Thr+H]^{+,}$ at *m/z* 120.2. At *m/z* 190.1, a product ion associated with the loss of -90 Da was observed. The IE standard experienced a neutral loss of 93 Da, 3 Da higher than the non-IE AA, indicating that one of the four ^{13}C of IE Thr was in the product ion at *m/z* 191.1 for the IE AA and *m/z* 190.1 for Thr.

<u>Piperonal:</u> showed dehydration at *m/z* 234.1 and an unidentified neutral loss of 88 Da, corresponding to *m/z* 164.1. This identical neutral loss was also observed in the case of <u>4-anisaldehyde</u>.

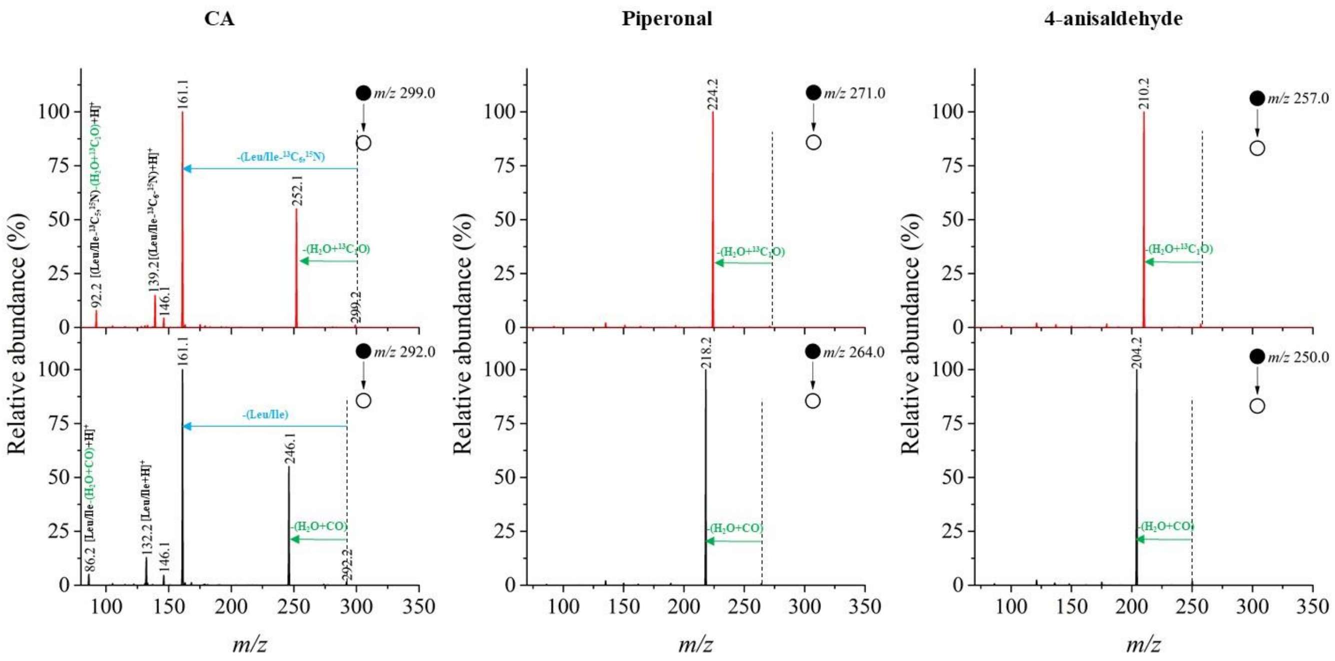

Figure S12. Tandem MS spectra obtained with the Thermo Finnigan LTQ linear ion trap for Leu/Ile (black trace) and Leu/Ile-$^{13}C_6$,^{15}N (red trace) after derivatization with CA, piperonal and 4-anisaldehyde.

Fragmentation of Leu/Ile:

<u>CA:</u> the tandem MS spectrum using CA displayed an initial loss of H_2O+CO detected at *m/z* 246.1, followed by the pseudo *b* ion corresponding to the loss of Leu/Ile (*m/z* 161.1), and the pseudo *y* ion, $[Leu/Ile+H]^+$, at *m/z* 132.2. Additionally, the product ion at *m/z* 86.2 was attributed to the loss of H_2O+CO from $[Leu/Ile+H]^+$.

<u>Piperonal and 4-anisaldehyde:</u> for both piperonal and 4-anisaldehyde, a SB was formed leading to fragmentation through the loss of -46 Da (H_2O+CO).

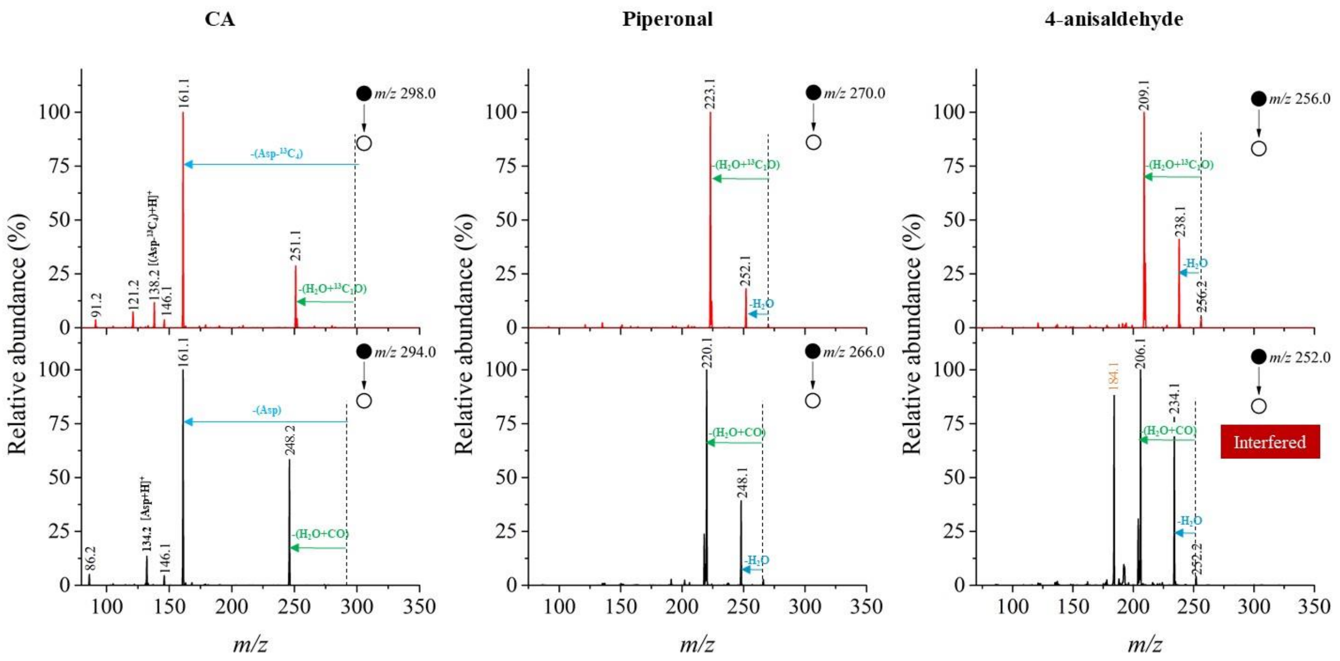

Figure S13. Tandem MS spectra obtained with the Thermo Finnigan LTQ linear ion trap for Asp (black trace) and Asp-$^{13}C_4$ (red trace) after derivatization with CA, piperonal and 4-anisaldehyde.

Fragmentation of Asp:

<u>CA:</u> in the case of CA, the tandem MS spectrum presented an initial loss of H_2O+CO at *m/z* 246.1, followed by the pseudo *b* ion corresponding to the loss of Asp (*m/z* 161.1), and the pseudo *y* ion, $[Asp+H]^+$, at *m/z* 134.2.

<u>Piperonal and 4-anisaldehyde:</u> both piperonal and 4-anisaldehyde led to SB formations that fragmented through the loss of H_2O (-18 Da) and -46 Da (H_2O+CO). Additionally, the Asp-4-anisaldehyde SB exhibited interference at *m/z* 184.1.

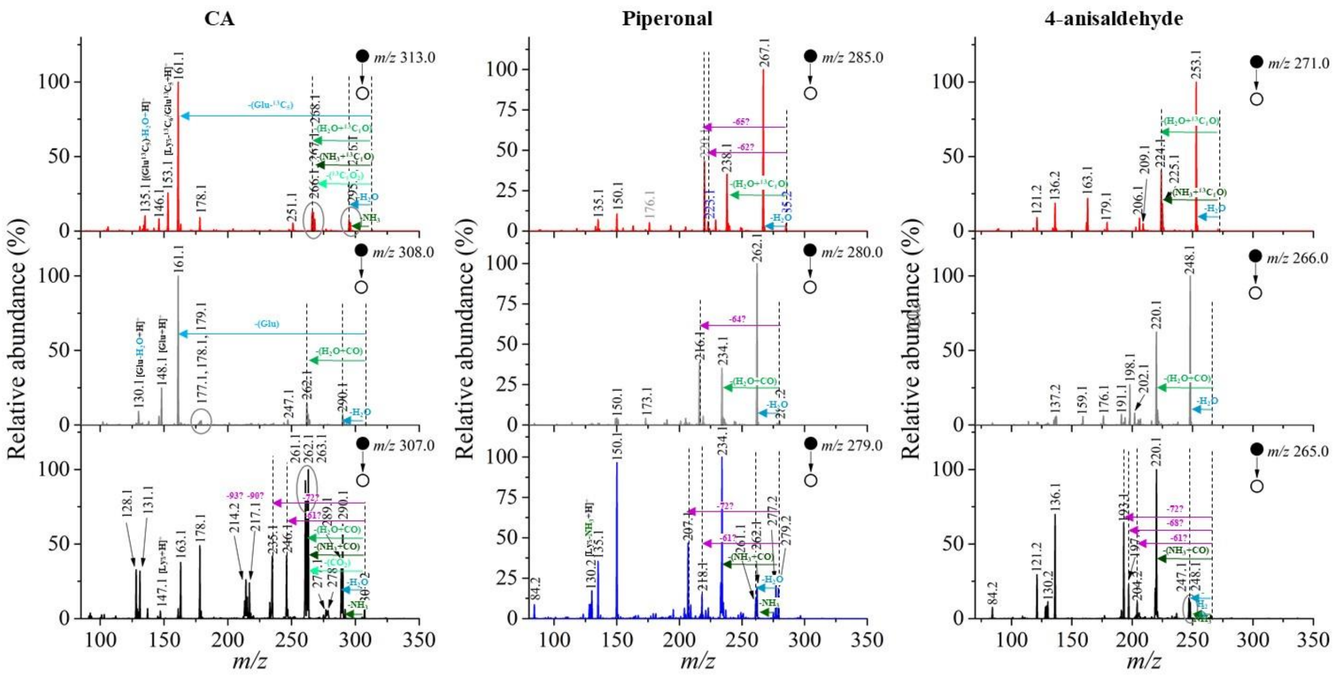

Figure S14. Tandem MS spectra obtained with the Thermo Finnigan LTQ linear ion trap for Lys (black trace), Glu (grey trace) and Lys-$^{13}C_6$+ Glu-$^{13}C_5$ (red trace) d after derivatization with CA, piperonal and 4-anisaldehyde.

The situation with Lys and Glu is complex due to the isobaric nature of their IE standards in the commercial mix for the resolving power of the ion trap. Consequently, the tandem MS spectra of *m/z* 313.0 combine the signals of both AAs.

<u>CA:</u> for CA, the tandem MS spectrum of Lys exhibited losses of NH_3 (-17 Da), CO_2 (-44 Da), NH_3+CO (-45 Da), and H_2O+CO (-46 Da), alongside the pseudo *b* ion detected at *m/z* 161.1 and pseudo *y* ion at *m/z* 147.1. Notably, Lys, with two primary amines, displays a higher likelihood for the ε-position to react. A neutral loss of -72 Da suggests potential imine formation via the α-amino and α-cleavage of the organic substituent. Other neutral losses, like 61, 90, and 93 Da, were not explicitly identified.

Conversely, tandem MS of Glu spectrum showcased simpler losses: H_2O detected at *m/z* 290.1, H_2O+CO at *m/z* 262.1, the pseudo *b* ion at *m/z* 161.1, the pseudo *y* ion at *m/z* 148.2, and the water loss from the latter at *m/z* 130.1. However, the *m/z* 313.0 tandem MS spectrum presented multiple product ions that potentially belong to either Lys or Glu, making specific assignments challenging due to their similar masses.

<u>Piperonal and 4-anisldehyde</u>: for piperonal and 4-anisaldehyde, both compounds caused the loss of NH_3, H_2O, and NH_3+CO for Lys, coupled with unannotated neutral losses like -61 Da and -72 Da. Notably, product ions related to the SB with the imine and substituted phenyl were observed at *m/z* 150.1 for piperonal and *m/z* 136.1 for 4-anisaldehyde.

In Glu, both piperonal and 4-anisaldehyde exhibited consistent losses of water (-18 Da) and H_2O+CO (-46 Da), with no distinct product ions detected in the *m/z* 313.0 tandem MS spectrum.

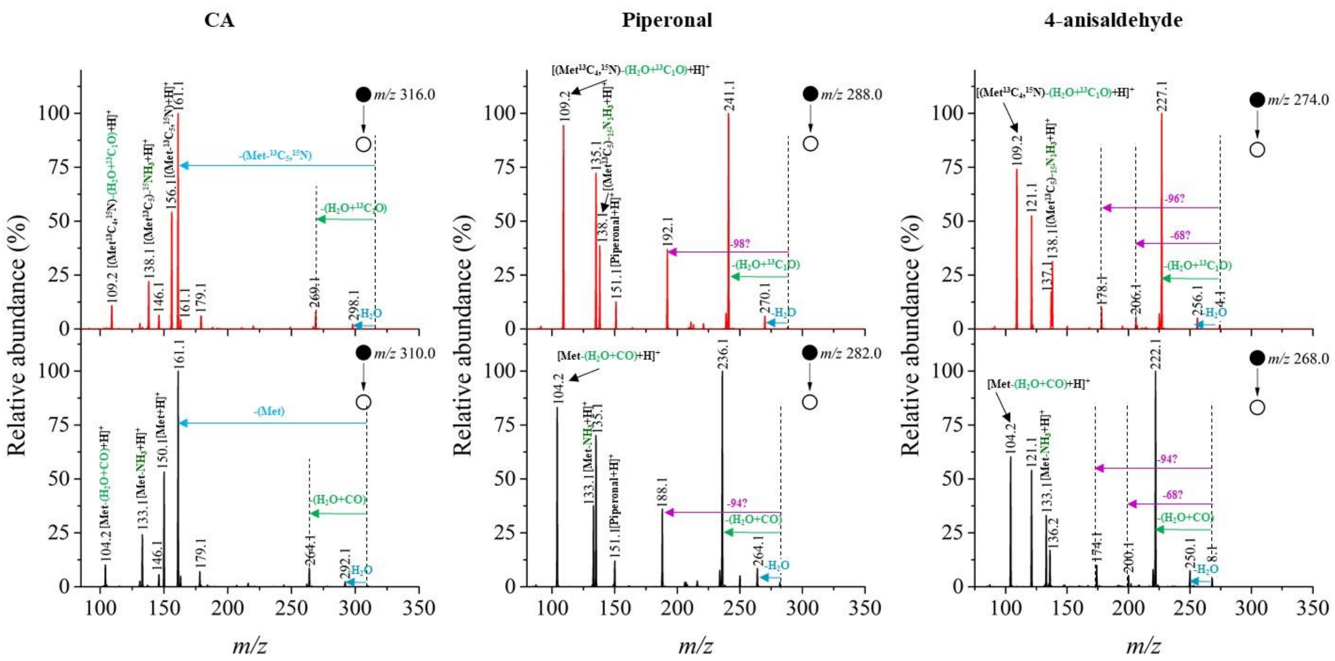

Figure S15. Tandem MS spectra obtained with the Thermo Finnigan LTQ linear ion trap for Met (black trace), and Met-$^{13}C_5$ (red trace) after derivatization with CA, piperonal and 4-anisaldehyde.

Fragmentation of Met:

The analysis of Met and His in real samples is not feasible with the IE AA mix used in the present work due to the isobaric nature of Met-$^{13}C_5$, ^{15}N and His. Nevertheless, in separate analyses of the AA mix and the IE AA mix, we characterized the spectra of both AAs.

<u>CA:</u> for CA, Met displayed water loss at *m/z* 292.1 and H_2O+CO at *m/z* 264.1. It exhibited a pseudo *b* ion at *m/z* 161.1, accompanied by the pseudo *y* ion at *m/z* 150.1, which also showcased losses of NH_3 at *m/z* 133.1 and H_2O+CO at *m/z* 104.2, confirmed by IE Met analysis.

<u>Piperonal and 4-anisaldehyde:</u> both piperonal and 4-anisaldehyde facilitated SB with Met that fragmented through losses of H_2O (-18 Da) and 46 Da (H_2O+CO). Furthermore, product ions for $[Met\text{-}NH_3+H]^+$ and $[Met\text{-}(H_2O+CO)+H]^+$ were detected in both cases. Once again, a different unidentified neutral loss (-94 Da) was observed.

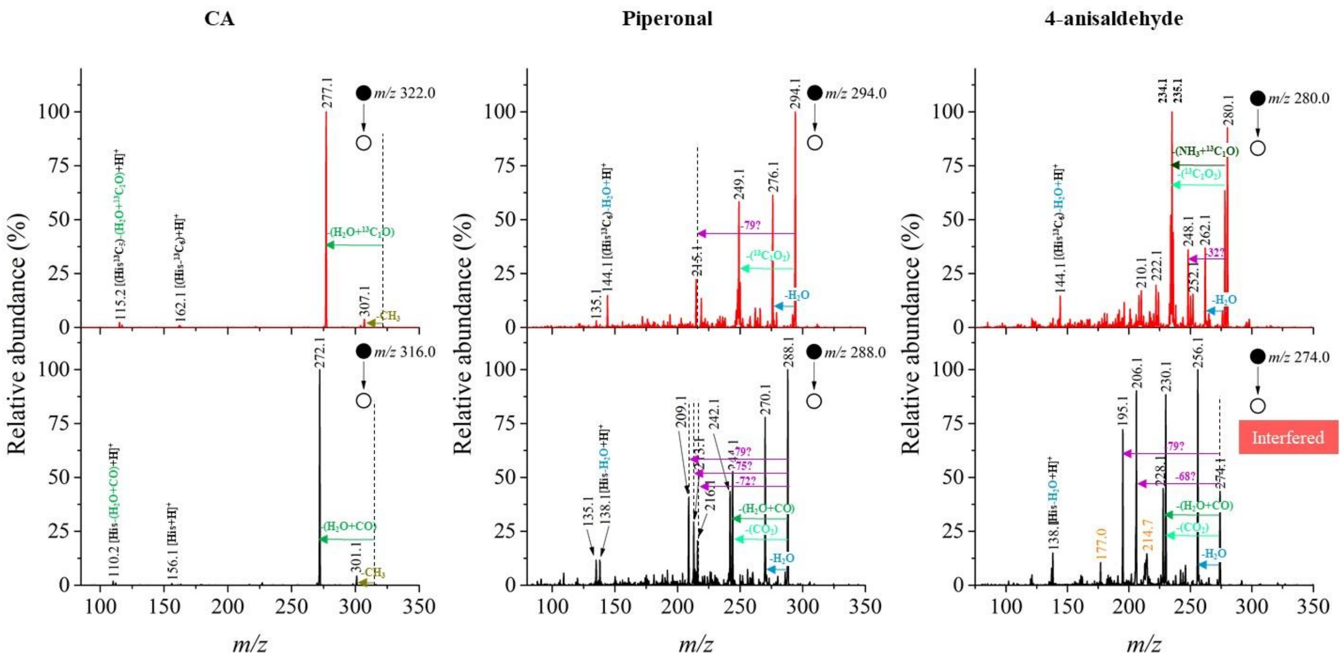

Figure S16. Tandem MS spectra obtained with the Thermo Finnigan LTQ linear ion trap for His (black trace), and His-$^{13}C_6$ (red trace) after derivatization with CA, piperonal and 4-anisaldehyde.

Fragmentation of His:

<u>CA:</u> His exhibited water loss observed at *m/z* 301.1 and H_2O+CO at *m/z* 272. The pseudo *y* ion appeared at *m/z* 156.1 and displayed the loss of H_2O+CO at *m/z* 110.2, validated by BIE Met analysis.

<u>Piperonal and 4-anisaldehyde:</u> His had notably lower conversion rates with these aldehydes. The spectra appeared noisier compared to CA. Still, we observed losses of H_2O (-18 Da), CO_2 (-44 Da), and 46 Da (H_2O+CO) accompanied by unidentified neutral losses such as -72, -75, and -79 Da for piperonal and -68 and -79 Da for 4-anisaldehyde. In both cases, the product ion for [His-H_2O+H]$^+$ was detected.

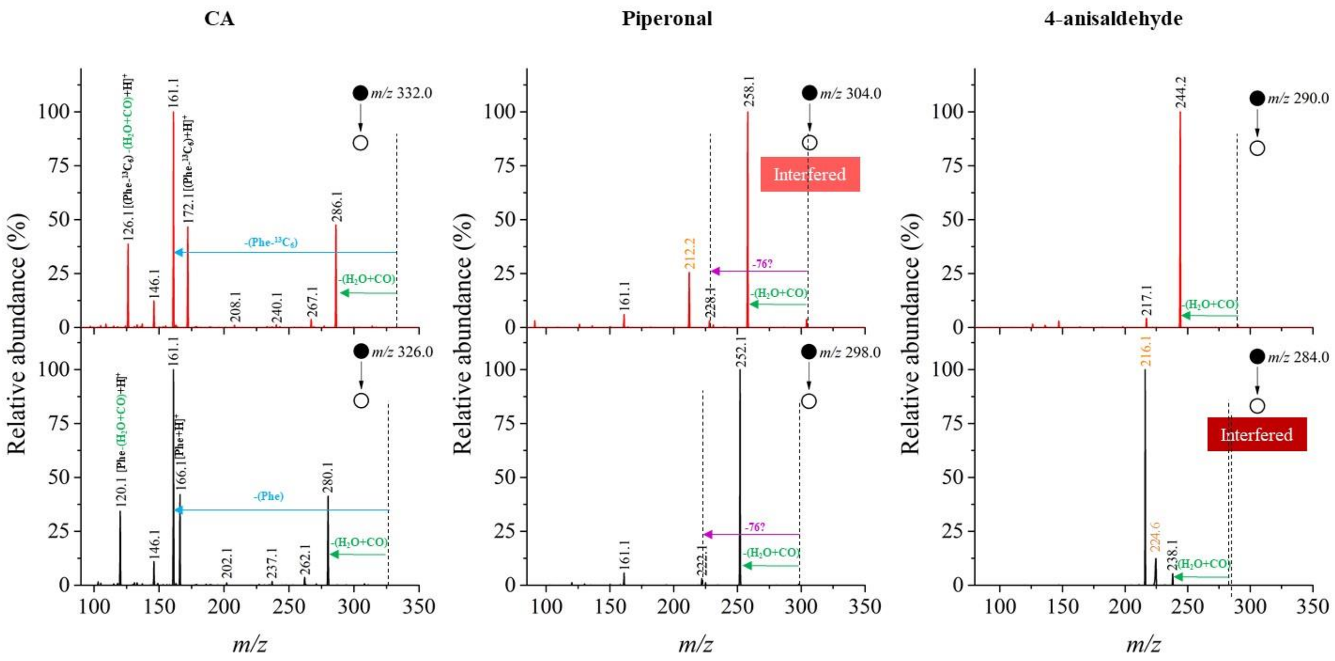

Figure S17. Tandem MS spectra obtained with the Thermo Finnigan LTQ linear ion trap for Phe (black trace), and Phe-$^{13}C_6$ (red trace) after derivatization with CA, piperonal and 4-anisaldehyde.

Fragmentation of Phe:

<u>CA:</u> the tandem MS spectrum of Phe exhibited a structurally rich profile with the detection of H_2O+CO loss at *m/z* 286.1. It presented the pseudo *b* ion at *m/z* 161.1 along with the pseudo *y* ion at *m/z* 166.1. The latter also displayed the loss of H_2O+CO at *m/z* 120.2, validated by IE Phe analysis.

<u>Piperonal and 4-anisaldehyde:</u> both aldehydes only showed the loss of 46 Da (H_2O+CO) from the precursor ion. Additionally, in case or the other the AA or the IE showed interferences hampering the identification.

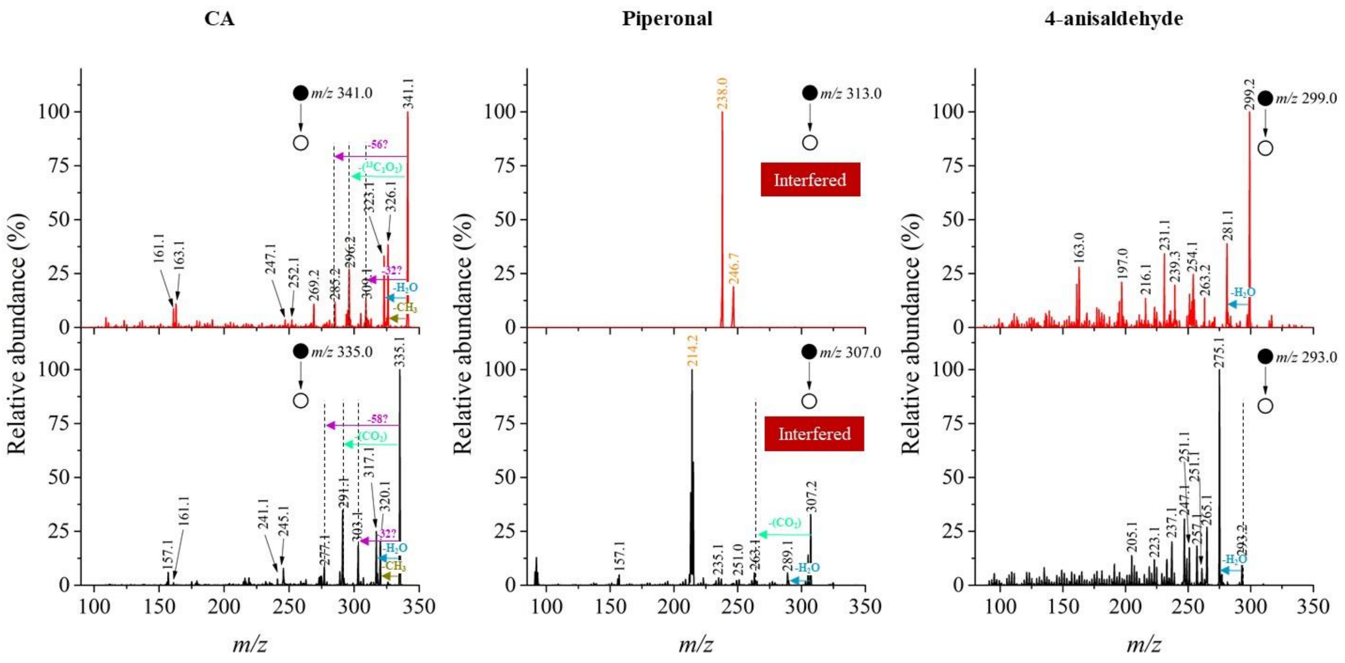

Figure S18. Tandem MS spectra obtained with the Thermo Finnigan LTQ linear ion trap for Arg (black trace), and Arg-$^{13}C_6$ (red trace) after derivatization with CA, piperonal and 4-anisaldehyde.

Fragmentation of Arg:

Arg is similar to Lys due to its two reaction sites: the amino group or the guanidinium. Annotating the tandem MS spectra proved complicated with ion trap results.

CA: Arg exhibited losses of CH_3 (-15 Da), H_2O (-18 Da), and CO_2 (-44 Da), alongside with the unidentified neutral losses of -32 Da and -58 Da.

Piperonal and 4-anisaldehyde: both compounds showed interference in the fragmentation process.

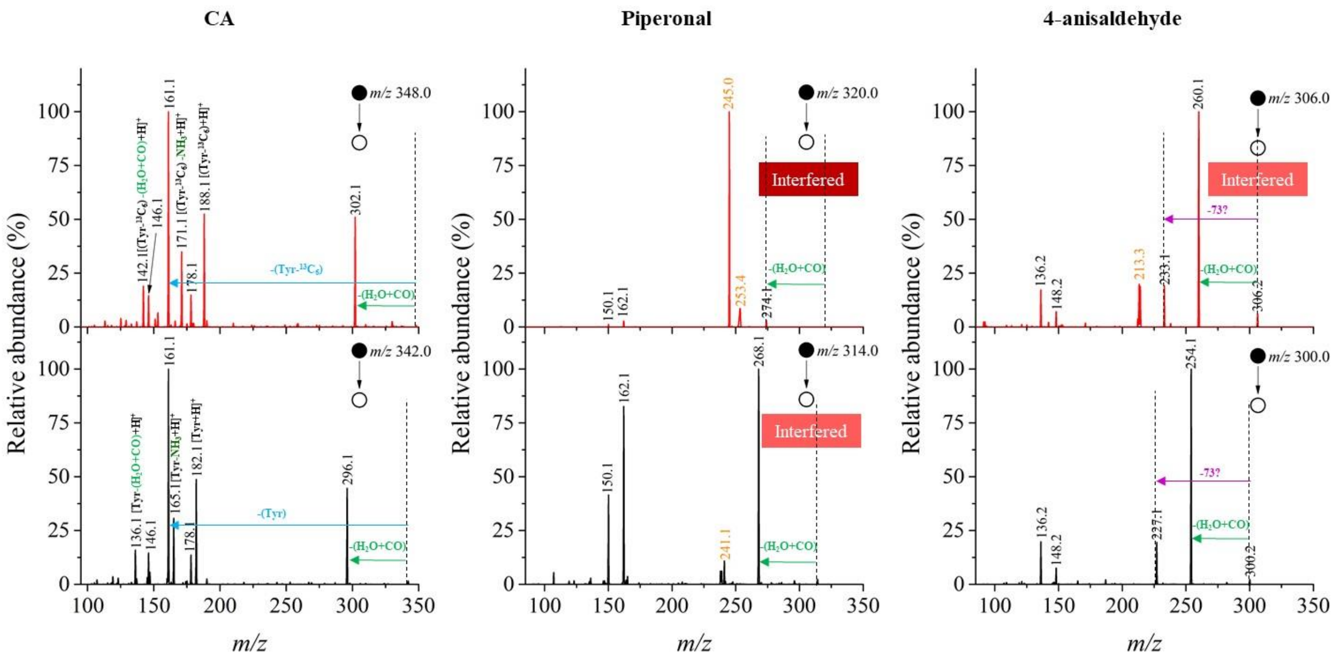

Figure S19. Tandem MS spectra obtained with the Thermo Finnigan LTQ linear ion trap for Tyr (black trace), and Tyr-$^{13}C_6$ (red trace) after derivatization with CA, piperonal and 4-anisaldehyde.

Fragmentation of Tyr:

CA: Tyr exhibited the loss of H_2O+CO, observed at *m/z* 296.1. The pseudo *b* ion appeared at *m/z* 161.1, accompanied by the pseudo *y* ion at *m/z* 182.1, which also displayed losses of NH_3 at *m/z* 165.1 and H_2O+CO at m/z 136. These observations were consistent with the analysis of IE Tyr.

Piperonal and 4-anisaldehyde: Both compounds resulted in interference, although the precursor ion showed the loss of 46 Da (H_2O+CO).

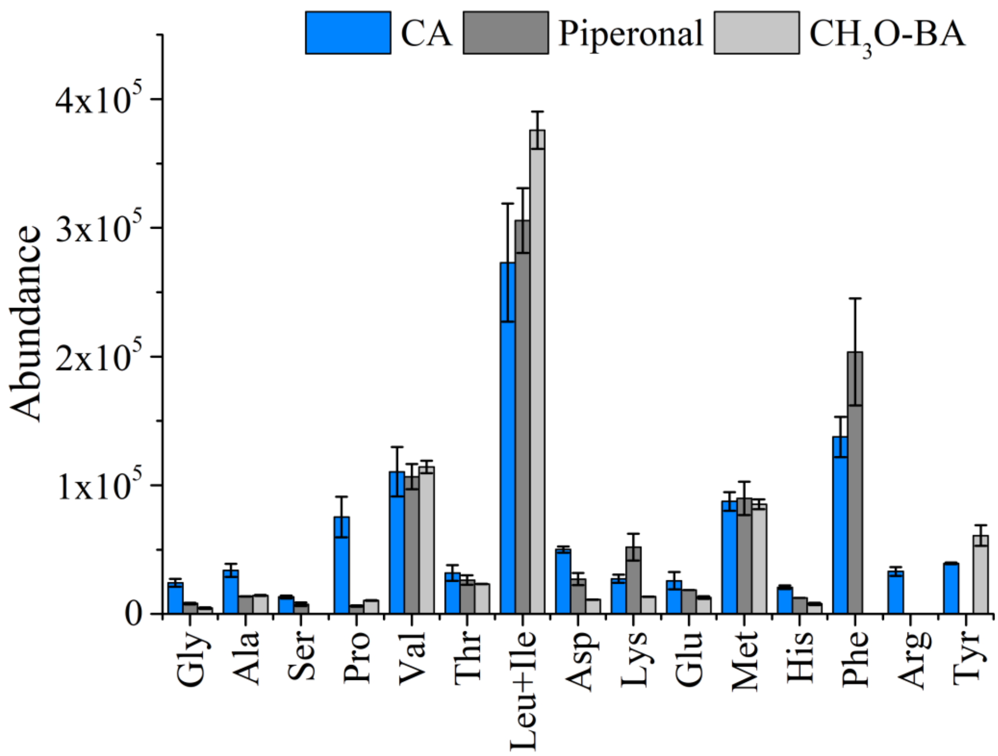

Figure S20. Comparison of abundances corresponding to the various SB formed during the derivatization of the commercial AA mixture (250 µM each). The samples were derivatized following the optimized method using three different agents: CA (in blue), piperonal (in dark gray), and 4-anisaldehyde or CH3O-BA (in light gray).

Table S1. Multiple reaction monitoring (MRM) parameters utilized for AA quantification via PS-MS utilizing the TSQ Quantiva mass spectrometer. MRM experiments were conducted using a collision gas pressure of 1.5 mTorr and a full width at half maximum (FWHM) window of 0.4 for Q1 and Q3.

**Compound**	**Retention Time (min)**	**Polarity**	**Precursor ion (*m/z*)**	**Product ion (*m/z*)**	**Collision Energy (V)**
Gly	0.5	Positive	236	161	16.42
Gly-IE	0.5	Positive	239	161	16.42
Ala	0.5	Positive	250	161	19.15
Ala-IE	0.5	Positive	254	161	19.15
Ser	0.5	Positive	266	161	18.7
Ser-IE	0.5	Positive	270	161	18.7
Pro	0.5	Positive	276	161	20.01
Val	0.5	Positive	278	161	21.73
Thr	0.5	Positive	280	161	18.65
Pro-IE	0.5	Positive	281	161	20.01
Val-IE	0.5	Positive	283	161	21.73
Thr-IE	0.5	Positive	284	161	18.65
Leu	0.5	Positive	292	161	22.54
Asp	0.5	Positive	294	161	19.56
Asp-IE	0.5	Positive	298	161	19.56
Leu-IE	0.5	Positive	299	161	22.54
Phe	0.5	Positive	326	161	22.29
Phe-IE	0.5	Positive	332	161	22.29
Arg	0.5	Positive	335	161	20.77
Arg-IE	0.5	Positive	341	161	20.77
Tyr	0.5	Positive	342	161	20.77
Tyr-IE	0.5	Positive	348	161	20.77

Table S2. Chromatographic conditions utilized for separation, employing phase A consisting of 80% H_2O/20% ACN, 10 mM ammonium formate, and 0.1% formic acid, and phase B comprising 100% ACN with 0.1% formic acid. The injection volume was 10 μL.

Time	Flow (mL/min)	%B	Curve
0	0.15	95	5
0.5	0.15	95	5
8	0.15	40	5
9.4	0.15	40	5
9.5	0.15	95	5
11	0.15	95	5
12	0.15	95	5

Table S3. a) Analysis parameters for LC-QqQ utilizing the TSQ Quantiva mass spectrometer. b) MRM parameters employed for AA quantification via LC-QqQ. MRM experiments were conducted with a collision gas pressure set at 1.5 mTorr, utilizing a 0.4 FWHM window for both Q1 and Q3.

a)

Parameter	Value
Spray voltage (V)	3500
Sheath gas (Arb)	35
Auxiliary gas (Arb)	10
Sweep gas (Arb)	0
Ion Transfer Tube Temp (ºC)	325
Vaporizer Temp (ºC)	275

b)

Compound	Retention Time (min)	Polarity	Precursor ion (*m/z*)	Product ion (*m/z*)	Collision Energy (V)
Gly	6.39	Positive	76	30	10.25
Gly-IE	6.39	Positive	79	32	10.25
Ala	6.16	Positive	90	44	10.25
Ala-IE	6.16	Positive	94	47	10.25
Ser	6.63	Positive	106	60	12.1
Ser-IE	6.63	Positive	110	63	12.1
Pro	5.69	Positive	116	70	13.54
Val	5.65	Positive	118	72	11.77
Thr	6.36	Positive	120	74	19.19
Pro-IE	5.69	Positive	121	74	13.54
Val-IE	5.65	Positive	123	76	11.77
Thr-IE	6.36	Positive	124	77	19.19
Leu	5.2	Positive	132	86	10.25
Asp	5.44	Positive	134	88	13.54
Asp-IE	5.44	Positive	138	91	13.54
Leu-IE	5.2	Positive	139	92	10.25
Phe	5.1	Positive	166	120	14.45
Phe-IE	5.1	Positive	172	126	14.45
Arg	7.32	Positive	175	130	10.21
Arg-IE	7.32	Positive	181	135	10.24
Tyr	5.66	Positive	182	136	14.85
Tyr-IE	5.66	Positive	188	142	14.85

Table S4. Figures of merit for LC-QqQ calibration curves: Intercept and Slope

AA	Intercept	ST	Slope	ST	R^2
**Gly**	-0.0820	0.0547	0.0648	0.0011	0.9961
**Ala**	-0.1807	0.0450	0.0894	0.0020	0.9933
**Ser**	-0.1688	0.0248	0.0792	0.0011	0.9974
**Pro**	-0.1575	0.0575	0.1077	0.0025	0.9925
**Val**	-0.1306	0.0261	0.1005	0.0011	0.9982
**Thr**	-0.1406	0.0245	0.0801	0.0011	0.9975
**Leu + Ile**	-0.1108	0.0178	0.0919	0.0008	0.9990
**Asp**	0.0003	0.0077	0.0040	0.0002	0.9797
**Lys**	-0.0231	0.0062	0.0147	0.0003	0.9953
**Glu**	-0.1636	0.0219	0.0873	0.0010	0.9983
**Met**	-0.1235	0.0181	0.0927	0.0008	0.9990
**His**	-0.0775	0.0181	0.0314	0.0008	0.9913
**Phe**	-0.1097	0.0188	0.0981	0.0008	0.9990
**Arg**	-0.0911	0.0208	0.0358	0.0009	0.9911
**Tyr**	-0.1371	0.0244	0.0964	0.0011	0.9983

Table S5. Accuracy comparison between the Premix strategy using Whatman 903 paper and the On-paper strategy using Whatman 1 paper for SB-PS-MS analysis of human plasma, with LC-MS quantification as the reference value.

AA	$[AA]_{Human\ Plasma\ analysis\ LC\text{-}MS}$ (µM)	$[AA]_{Human\ PreMix\ Whatman\ 903\ PS\text{-}MS}$ (µM)	%Bias $PreMix_{Whatman\ 903}$-LC	$[AA]_{On\ paper\ Whatman\ 1\ PS\text{-}MS}$ (µM)	%Bias On $paper_{Whatman\ 1}$– LC
**Gly**	156	125	20%	172	10%
**Ala**	153	202	31%	266	74%
**Ser**	90.1	748	730%	122	35%
**Pro**	89.5	53	41%	128	43%
**Val**	93.3	107	15%	156	67%
**Thr**	93.1	557	498%	197	111%
**Leu + Ile**	57.2	62.3	9%	82.2	44%
**Asp**	-	73.8	-	23.2	-
**Phe**	35.3	38.1	8%	42.5	20%
**Arg**	152	88.1	42%	198	31%
**Tyr**	43.7	58.8	35%	50.4	15%
